# The epilepsy and intellectual disability-associated protein TBC1D24 regulates the maintenance of excitatory synapses and animal behaviors

**DOI:** 10.1371/journal.pgen.1008587

**Published:** 2020-01-31

**Authors:** Lianfeng Lin, Quanwei Lyu, Pui-Yi Kwan, Junjun Zhao, Ruolin Fan, Anping Chai, Cora Sau Wan Lai, Ying-Shing Chan, Xuting Shen, Kwok-On Lai

**Affiliations:** 1 School of Biomedical Sciences, The University of Hong Kong, Hong Kong, China; 2 State Key Laboratory of Brain and Cognitive Sciences, The University of Hong Kong, Hong Kong, China; Columbia University Medical Center, UNITED STATES

## Abstract

Perturbation of synapse development underlies many inherited neurodevelopmental disorders including intellectual disability (ID). Diverse mutations on the human *TBC1D24* gene are strongly associated with epilepsy and ID. However, the physiological function of TBC1D24 in the brain is not well understood, and there is a lack of genetic mouse model that mimics TBC1D24 loss-of-function for the study of animal behaviors. Here we report that TBC1D24 is present at the postsynaptic sites of excitatory synapses, where it is required for the maintenance of dendritic spines through inhibition of the small GTPase ARF6. Mice subjected to viral-mediated knockdown of TBC1D24 in the adult hippocampus display dendritic spine loss, deficits in contextual fear memory, as well as abnormal behaviors including hyperactivity and increased anxiety. Interestingly, we show that the protein stability of TBC1D24 is diminished by the disease-associated missense mutation that leads to F251L amino acid substitution. We further generate the F251L knock-in mice, and the homozygous mutants show increased neuronal excitability, spontaneous seizure and pre-mature death. Moreover, the heterozygous F251L knock-in mice survive into adulthood but display dendritic spine defects and impaired memory. Our findings therefore uncover a previously uncharacterized postsynaptic function of TBC1D24, and suggest that impaired dendritic spine maintenance contributes to the pathophysiology of individuals harboring *TBC1D24* gene mutations. The F251L knock-in mice represent a useful animal model for investigation of the mechanistic link between *TBC1D24* loss-of-function and neurodevelopmental disorders.

## Author summary

Neurons in the brain process and store information via the specialized structures called synapses. Excitatory synapses are located on the dendritic structures called dendritic spines. Despite its small size, each dendritic spine contains a highly complicated signaling network consisting of enormous number of proteins. It is believed that dysfunction of these proteins will affect synaptic transmission and causes cognitive deficits that underlie neurodevelopmental disorders such as intellectual disability (ID). Recently many novel ID-associated gene mutations have been identified. The next crucial step will be to delineate how the gene products function normally in the brain, which will lead to better understanding of the pathophysiology of ID. Human genetic studies have linked numerous mutations on *TBC1D24*, a gene whose function is not well-defined, to epilepsy and intellectual disability. Here we show that TBC1D24 is localized at excitatory synapses of neurons in the hippocampus, a brain region that is critical to learning and memory. By knocking down TBC1D24 expression through RNA interference or generation of transgenic mice harboring an ID-associated *Tbc1d24* missense mutation that destabilizes the protein, we demonstrate that TBC1D24 deficiency impairs the maintenance of dendritic spines in the brain as well as memory formation. This study indicates that synaptic defects underlie the cognitive deficits of individuals carrying *TBC1D24* gene mutations.

## Introduction

Disrupted development of neuronal synapses is a common cause of diverse brain disorders [1–3]. The majority of excitatory synapses in mammalian central nervous system are present on dendritic spines [4]. Proteomic studies reveal an unexpectedly large number of proteins present in the postsynaptic density (PSD) of excitatory synapses [5]. Many of these are signaling molecules such as small GTPases, which orchestrate the formation, maturation and maintenance of dendritic spines [6–8]. Because their binding affinity to downstream effector targets is much higher in the GTP-bound conformation, the activity of each GTPase is in turn regulated by multiple guanine nucleotide exchange factors (GEFs) and GTPase-activating proteins (GAPs). Genetic variations that alter GTPase signaling due to dysfunction of GEFs and GAPs, and the subsequent defect in dendritic spine morphogenesis, often underlie neurological disorders such as autism spectrum disorders (ASD), schizophrenia and intellectual disability (ID) [9]. Deviation of the balance between excitatory and inhibitory synapses also perturbs neural circuit function that may result in neurological manifestations such as epilepsy, a common comorbidity with ASD and ID. Elucidating the corresponding GTPase function and understanding how they are regulated in neuron are therefore crucial for identifying therapeutic molecular targets [10].

Wide spectrum of missense and frameshift mutations have been identified on the *TBC1D24* gene in about 50 patients with syndromic ID and the deafness, onychodystrophy, osteodystrophy, mental retardation, and seizures (DOORS) syndrome, both of which are associated with different types of epilepsy [11]. TBC1D24 belongs to the TBC/RAB family of GAP proteins which contain the characteristic Tre2/Bub2/Cdc16 (TBC) domain. While there are more than 40 TBC domain-containing proteins, TBC1D24 is unusual because it also possesses a TBC-LysM (TLDc) domain with unknown function. In addition, TBC1D24 is an unconventional TBC/RABGAP because it lacks the "arginine finger" in the TBC domain and therefore may catalyze the GTP hydrolysis of its downstream target in an alternative way [12]. Besides Rab-GTPases, ectopically expressed TBC1D24 also binds and suppresses the activity of ARF6 [13], a small GTPase of the Ras superfamily which plays a key role in the control of vesicular traffic and actin organization at the cell periphery [14].

TBC1D24 is highly expressed in the human brain [13]. The presence of non-sense mutations suggests that loss-of-function is detrimental, but the physiological function of TBC1D24 has only begun to be elucidated. In *Drosophila*, the TBC1D24 ortholog, skywalker (Sky), is present at the presynaptic terminal of the neuromuscular junction, where it regulates neurotransmitter release by modulating vesicular trafficking between the endosomes and lysosomes [15, 16]. In mouse, TBC1D24 is crucial for neuronal migration in the cerebral cortex during early development [17]. However, information on TBC1D24 function in the mature rodent brain *in vivo* is limited. Moreover, whether disease-related *tbc1d24* mutations in experimental animals impair cognitive functions such as learning and memory that are relevant to ID has not been explored.

Besides regulating synaptic vesicle recycling and neurotransmitter release at the presynaptic terminal [18], the putative TBC1D24 downstream target ARF6 also controls AMPA receptor trafficking and development of dendritic spines [19–24]. Since perturbed excitatory PSD signaling is linked to both epilepsy and ID [5, 25, 26], we aim to explore the postsynaptic function of TBC1D24. Here we report that TBC1D24 is present in the PSD of primary hippocampal neuron, and it maintains dendritic spines partly through suppression of ARF6. Neurons with TBC1D24 deficiency caused by shRNA-mediated knockdown or an ID-associated F251L missense mutation [27, 28] exhibit postsynaptic defects; while acute knockdown of TBC1D24 in the hippocampus or heterozygous F251L mutation leads to memory impairment. Therefore, in addition to presynaptic neurotransmitter release, our findings reveal that TBC1D24 also plays a key role in the maintenance of glutamatergic synapses of the postsynaptic neuron. Alteration of excitatory synapses due to loss of TBC1D24 expression may underlie the pathogenesis of *TBC1D24* F251L mutation-associated intellectual disability.

## Results

### TBC1D24 is present at excitatory synapses of postsynaptic hippocampal neuron

**Fig 1:**
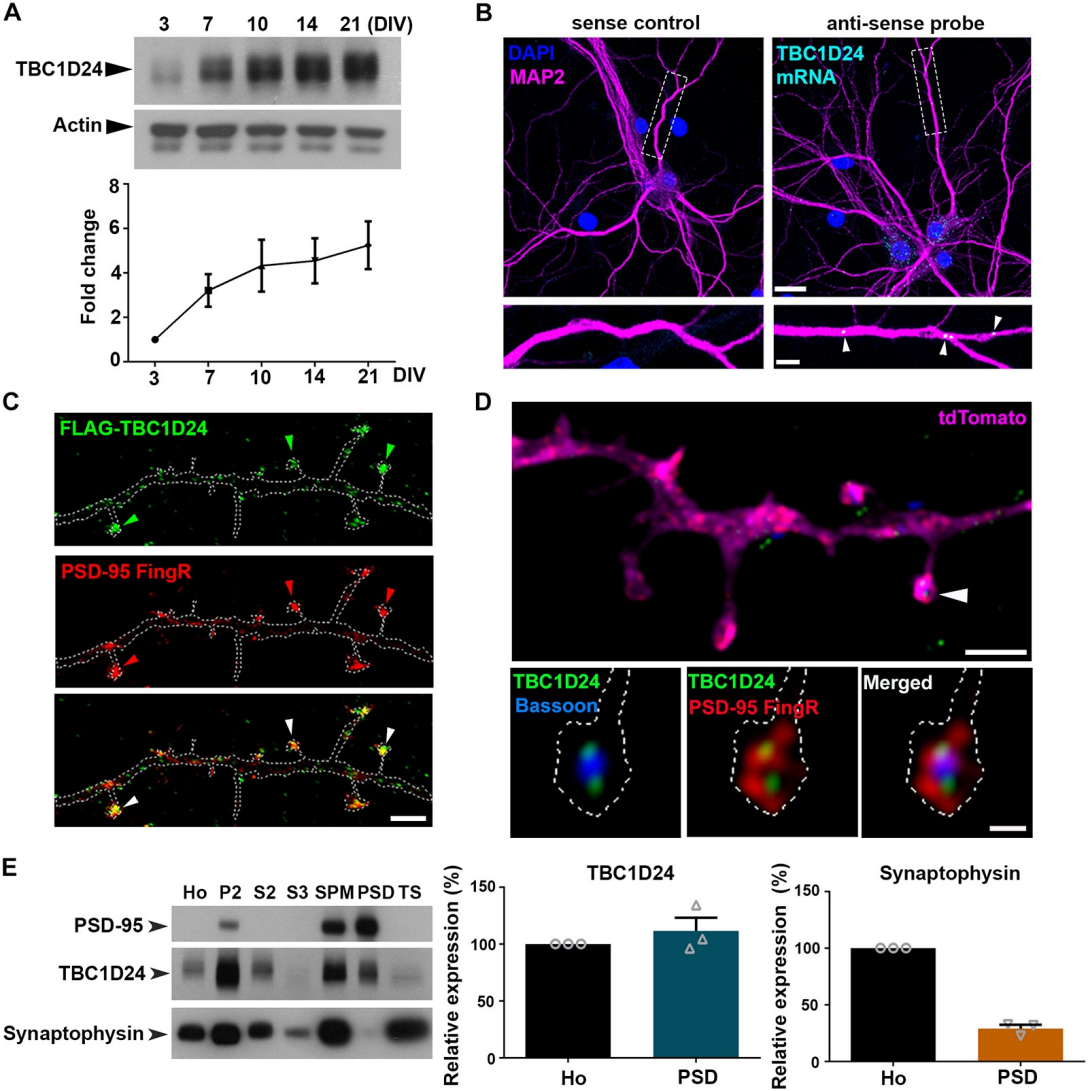
TBC1D24 is present at the postsynaptic site of excitatory synapse. **(A)** Expression of TBC1D24 in cultured hippocampal neuron was increased upon neuronal maturation. The duration of culture was indicated as *days in vitro* (DIV). Results were pooled from three independent experiments. **(B)** *In situ* hybridization was performed on hippocampal neurons (17 DIV), followed by immunofluorescence staining of MAP2 to label the dendrites. *Tbc1d24* mRNA granules (cyan, arrowheads) were detected in dendrites (magenta). Specificity of signal was verified by the absence of mRNA puncta after hybridization with sense probe. Scale bars: top, 20 μm; bottom, 5 μm. **(C)** Hippocampal neurons were co-transfected with FLAG-TBC1D24, GFP-tagged PSD-95-FingR intrabody and tdTomato. Immunocytochemistry (17 DIV) with antibodies against FLAG, GFP and tdTomato confirmed the synaptic localization of FLAG-TBC1D24 in dendritic spines (arrowheads). Scale bar: 2 μm. **(D)** Hippocampal neurons were co-transfected with GFP-tagged PSD-95-FingR and tdTomato. At 18 DIV, immunostaining was performed with antibodies against TBC1D24, the presynaptic marker Bassoon and GFP. Images were taken with super-resolution SIM. Scale bars: top, 1 μm; bottom, 0.2 μm. **(E)** Western blot showed the abundance of TBC1D24 but not synaptophysin in the PSD (postsynaptic density) fraction of adult mouse brain (n = 3). Ho, crude homogenate; P2, synaptosomal fraction; S2, cytosol/light membranes; S3, crude synaptic vesicle; SPM, synaptic plasma membrane; TS, Triton X-100 soluble fraction.

To elucidate the function of TBC1D24, we first determined its expression profile and subcellular localization in cultured hippocampal neuron. We focus on the hippocampus because of high TBC1D24 expression in this brain area [13] and its importance in learning and memory, which is relevant since *TBC1D24* mutations are associated with intellectual disability. Although TBC1D24 regulates the migration of cortical neurons during embryonic stages [17], we found that the expression of TBC1D24 was largely up-regulated upon maturation of hippocampal neurons (Fig 1A), suggesting that its function extends beyond embryonic development. I*n situ* hybridization revealed the presence of *Tbc1d24* mRNA not only in the soma but also in distal dendrites (Fig 1B). Given that many dendritically localized transcripts encode postsynaptic proteins for synaptic transmission and plasticity [29, 30], we examined the subcellular localization of TBC1D24 protein in neuronal dendrites and synapses. Exogenously expressed FLAG-TBC1D24 clustered as puncta in both the dendritic shaft and dendritic spine heads of dissociated hippocampal neurons. Many of the puncta in the spine heads were co-localized with the excitatory postsynaptic protein PSD-95 (Fig 1C). Presence of endogenous TBC1D24 in the spine heads was confirmed by immunofluorescence staining using TBC1D24 antibody, the specificity of which has been verified by short hairpin RNA (shRNA) (S1A and S1B Fig). Super-resolution structured illumination microscopy (SR-SIM), which has about two fold higher optical resolution than conventional confocal microscope [31], was performed after triple staining with antibodies against TBC1D24, the presynaptic scaffold protein Bassoon, and GFP on neurons co-transfected with tdTomato and GFP-tagged PSD-95-FingR intrabody [32]. Quantification by Mander’s overlap coefficient indicated that about half of the TBC1D24 puncta on spine heads (n = 22) showed less co-localization with the Bassoon puncta than that with PSD-95 (Fig 1D), indicating that endogenous TBC1D24 on dendritic spine head is not entirely attributed to its localization at the presynaptic terminal but also postsynaptic expression. Fractionation of adult mouse brain indeed confirmed the presence of TBC1D24 in the postsynaptic density (PSD) (Fig 1E). Together these findings indicate that, in addition to the previously reported TBC1D24 localization at the presynaptic terminals [15, 16], TBC1D24 is also expressed at postsynaptic sites of excitatory synapses.

### TBC1D24 regulates the maintenance of dendritic spines and excitatory synapses in cultured hippocampal neuron *in vitro*

**Fig 2:**
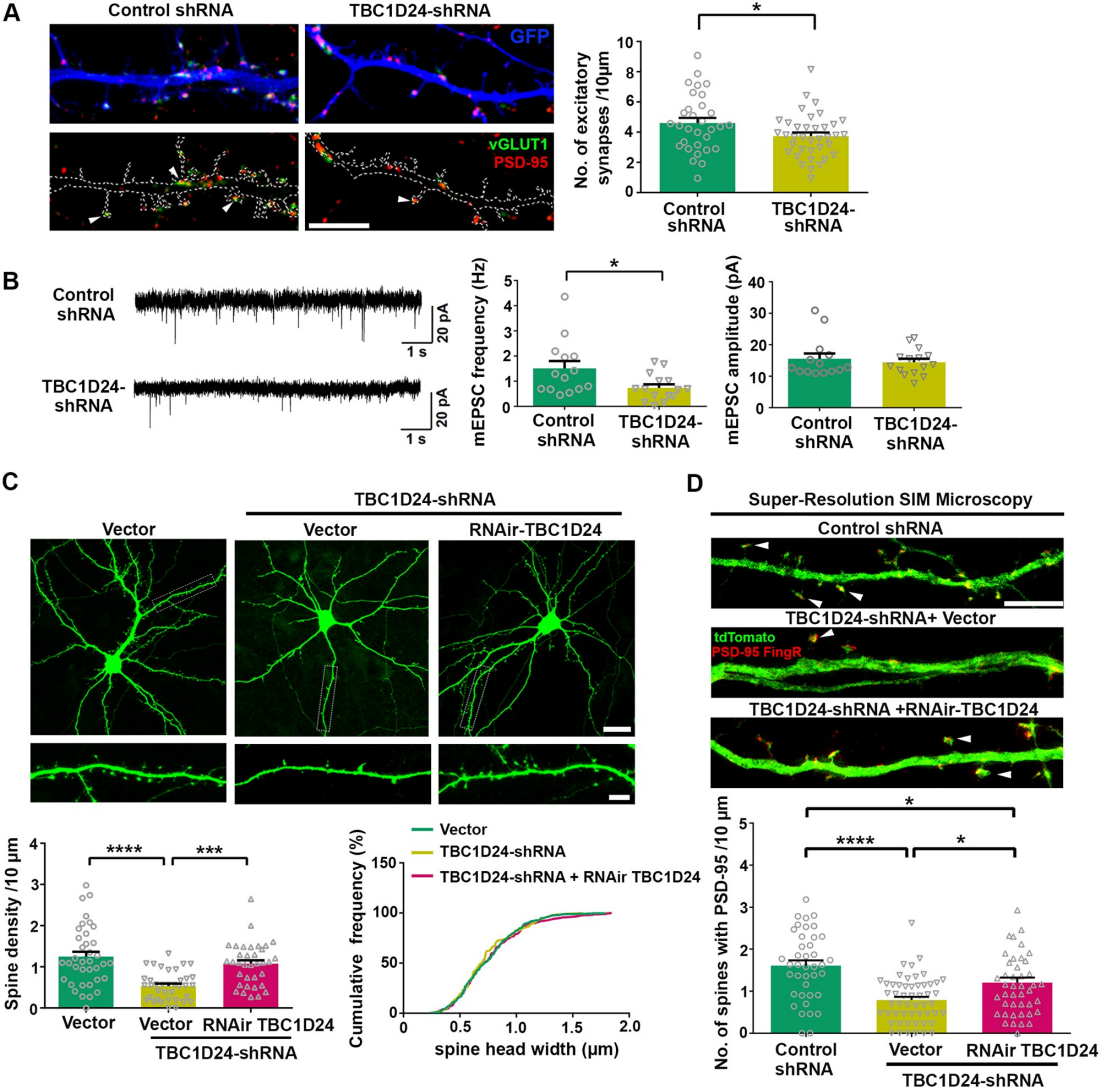
TBC1D24 is required for the maintenance of dendritic spines and excitatory synapses *in vitro*. **(A)** Hippocampal neurons (16 DIV) were co-transfected with GFP plasmid and scrambled shRNA (control) or shRNA targeting TBC1D24. Neurons were co-stained by antibodies against the excitatory synaptic markers vGLUT1 and PSD-95 at 3 days post-transfection. Density of excitatory synapses in dendrites was reduced in neurons expressing TBC1D24-shRNA (31–37 dendrites for each condition from three independent experiments; mean+SEM; *p<0.05; unpaired Student’s *t*-test). Scale bar: 5 μm. **(B)** Hippocampal neurons were transfected with scrambled shRNA or TBC1D24 shRNA at 15–16 DIV. Whole cell patch-clamp recording was performed 3 days post transfection in the presence of TTX and bicuculline. Representative traces were shown. Knockdown of TBC1D24 significantly decreased the frequency but not amplitude of miniature excitatory postsynaptic current (mEPSC) (14–15 neurons pooled from three independent experiments were recorded for each condition; mean+SEM; *p<0.05; Mann-Whitney test). **(C)** Representative images of hippocampal neurons transfected with GFP together with vector, TBC1D24-shRNA, or TBC1D24-shRNA plus RNAi-resistant TBC1D24. Neurons were transfected at 15 DIV and fixed three days after transfection. The spine loss induced by TBC1D24-shRNA was rescued by co-expression of RNAi-resistant TBC1D24 (35–37 dendrites from two independent experiments for each condition; mean+SEM; ***p<0.001, ****p<0.0001; one-way ANOVA followed by Tukey analysis). Scale bars: top, 20 μm; bottom, 5 μm. No significant difference in the width of dendritic spine heads was observed between different experimental conditions (126–285 spines from two independent experiments were analyzed for each condition). **(D)** Representative super-resolution SIM images of hippocampal neurons transfected with GFP together with control shRNA, TBC1D24-shRNA, or TBC1D24-shRNA plus RNAi-resistant TBC1D24. Neurons were transfected at 15 DIV and fixed three days after transfection. Loss of dendritic spines containing PSD-95 was induced by TBC1D24-shRNA, which was reversed by co-expressing RNAi-resistant TBC1D24 (data was pooled from two independent experiments, with 40–53 dendrites quantified for each condition; mean+SEM; *p<0.05, ****p<0.0001; Kruskal-Wallis test followed by Dunn’s multiple comparisons). Scale bar: 5 μm.

To determine the function of TBC1D24 on postsynaptic neuron, we introduced either the shRNA that targeted TBC1D24 or control shRNA into dissociated hippocampal neurons together with GFP construct, which outlined the dendrites and dendritic spines of the transfected neurons. The vast majority of neurons being analyzed are excitatory neurons since the percentage of GABAergic neurons in hippocampal cultured neurons is low [33, 34] (S1C Fig). Compared to neurons transfected with control shRNA, knockdown of TBC1D24 significantly reduced the number of excitatory synapses, as indicated by PSD-95 puncta that were juxtaposed to vGLUT1, on the GFP-positive neuron (Fig 2A). Moreover, the frequency but not the amplitude of miniature excitatory postsynaptic current (mEPSC) was reduced after knockdown of TBC1D24 (Fig 2B). The decrease in mEPSC frequency can be attributed to change in either neurotransmitter release or number of functional synapses. Since neurons were transfected using calcium phosphate precipitation which has very low transfection efficiency, the reduced mEPSC frequency of the GFP-positive postsynaptic neuron was likely resulted from cell-autonomous decrease in synapse number on the postsynaptic neuron, which was consistent with the observed fewer number of excitatory synapses (Fig 2A). Knockdown of TBC1D24 also significantly reduced the number of dendritic spines (Fig 2C and S1B Fig). The decrease in spine density was rescued by co-expression of the RNAi-resistant TBC1D24, thereby ruling out off-target effect. No change in the width of dendritic spine head was observed under different experimental conditions (Fig 2C). To rule out the possibility that the reduction of spine density is an artifact of co-transfecting two different plasmids, shRNA was generated using a vector containing GFP coding sequence, and similar decrease in spine density was observed upon introduction of the single TBC1D24-shRNA plasmid (S2 Fig). To confirm the effect of TBC1D24 deficiency on spine synapses, neurons were co-transfected with tdTomato and the GFP-tagged PSD-95 intrabody together with the shRNAs. Compared to control shRNA, the introduction of TBC1D24-shRNA significantly reduced the number of dendritic spines that contained PSD-95, and the synaptic loss was rescued by co-expression of RNAi-resistant TBC1D24 (Fig 2D). Since the shRNA was transfected into mature neurons when spines and synapses have already formed, our findings indicate that TBC1D24 is essential for maintaining the number of dendritic spines and excitatory synapses on postsynaptic neurons *in vitro*.

### TBC1D24 regulates dendritic spine maintenance through the suppression of ARF6 activity

**Fig 3:**
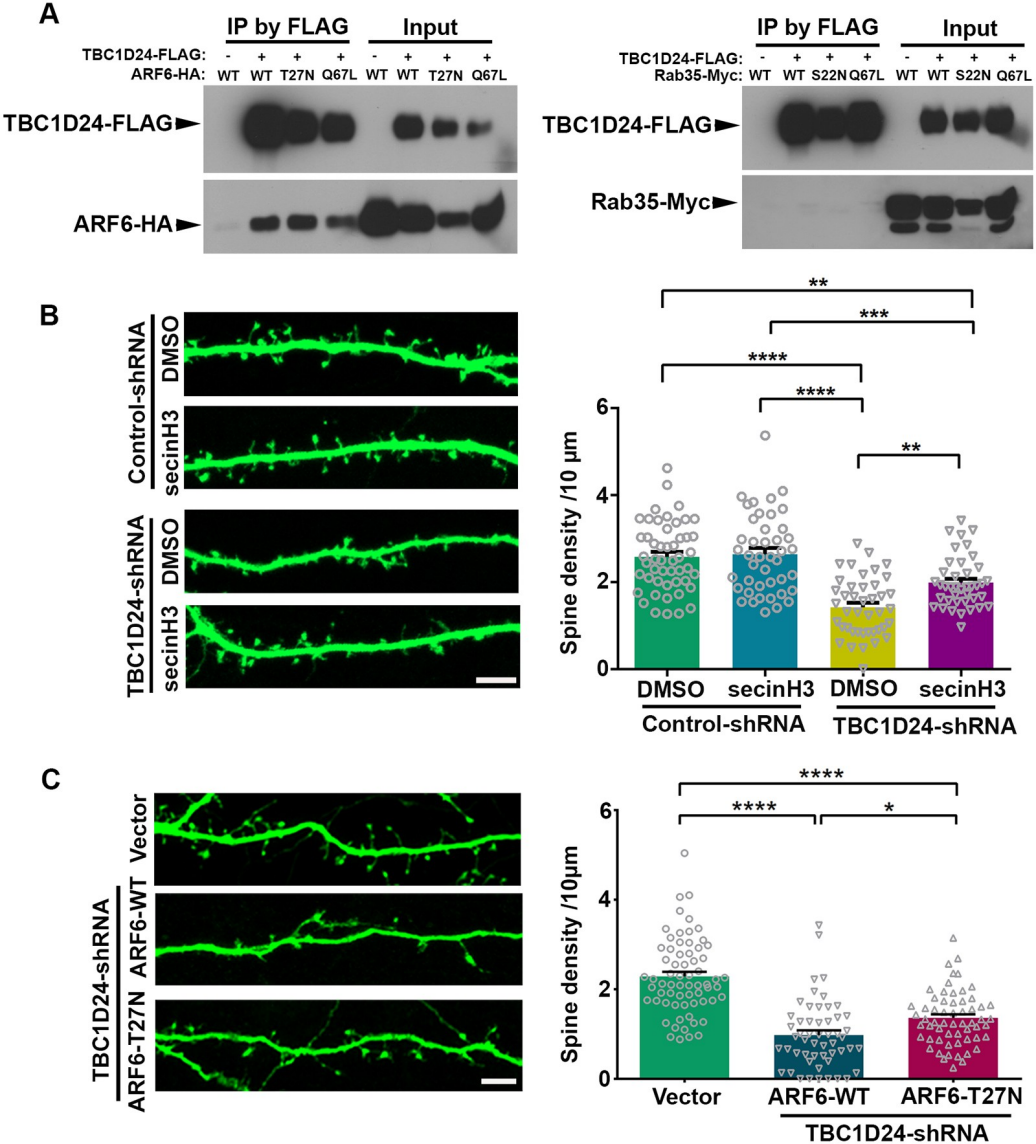
TBC1D24 maintains dendritic spines through suppression of ARF6. **(A)** HEK 293T cells were co-transfected with FLAG-tagged TBC1D24 plasmid together with HA-tagged ARF6 or Myc-tagged Rab35 wild-type (WT) or mutant constructs. Immunoprecipitation by FLAG beads revealed interaction of ARF6, but not Rab35, with TBC1D24. **(B)** Hippocampal neurons (15 DIV) were co-transfected with GFP and TBC1D24-shRNA or control shRNA, followed by treatment with secinH3 (30 μM) or DMSO (as vehicle control) for 6 hours at 3 days post transfection. SecinH3 reversed the TBC1D24-shRNA-induced reduction of dendritic spine density (40–50 dendrites from two independent experiments were quantified for each condition; mean+SEM; **p<0.01, ***p<0.001, ****p<0.0001; one-way ANOVA followed by Tukey analysis). Scale bars: 5 μm. **(C)** Hippocampal neurons (14–15 DIV) were co-transfected with GFP and control vector or TBC1D24-shRNA with or without wild-type (WT) or dominant-negative (T27N) ARF6. Neurons were fixed and immunostained with GFP antibody 3 days post transfection. The decrease in spine density after TBC1D24 knockdown was partially rescued by dominant-negative ARF6 (53–66 dendrites from three independent experiments for each condition; mean+SEM; *p<0.05, ****p<0.0001; Kruskal-Wallis test followed by Dunn’s multiple comparisons).

We next address the mechanism by which TBC1D24 maintains dendritic spines. In *Drosophila*, TBC1D24 regulates neurotransmitter release through modulation of Rab35 [16]. However, over-expression of TBC1D24 suppresses ARF6 activity in Hela cells [13], and ARF6 is implicated as the downstream target of TBC1D24 in mammalian neuron [17, 35]. We found that the mammalian TBC1D24 interacts with ARF6 but not Rab35 (Fig 3A). Furthermore, the constitutively-active ARF6 (Q67L) [36], but not the wild-type, dominant-negative ARF6 (T27N) or the mildly active ARF6 T157A mutant [37], significantly reduced the number of vGLUT1/PSD-95 puncta of hippocampal neurons (S3A Fig). We therefore hypothesize that TBC1D24 might mediate its synaptic function through inhibition of ARF6. Towards this end, we ask whether inhibiting ARF6 activity can rescue the reduction of spine density after depletion of TBC1D24. Hippocampal neurons were transfected with control- or TBC1D24-shRNA and treated with SecinH3, an inhibitor of the cytohesin subfamily of Arf-GEFs including that of ARF6 [38]. The dose of secinH3 in treating dissociated hippocampal neuron (30μM) followed that of previous study, which effectively reduces ARF6 activity in cultured hippocampal neuron [18]. SecinH3 treatment alone did not affect dendritic spine density but significantly reduced synapse number, which might be resulted from its additional effect on synapses on dendritic shaft and presynaptic terminals. Interestingly, secinH3 treatment partially reversed the reduction of dendritic spines and excitatory synapses induced by TBC1D24-shRNA (Fig 3B and S3B Fig). The role of ARF6 was further investigated by co-expressing dominant-negative (T27N) or wild-type ARF6 with the TBC1D24-shRNA. While over-expression of wild-type or dominant-negative ARF6 alone did not affect dendritic spines (S3C Fig), co-expression of dominant-negative ARF6 partially rescued the decrease in spine density induced by TBC1D24-shRNA (Fig 3C). These findings therefore indicate that TBC1D24 maintains dendritic spines in part through suppression of ARF6 activity.

### TBC1D24 is required for the maintenance of dendritic spines in adult hippocampus *in vivo*

**Fig 4:**
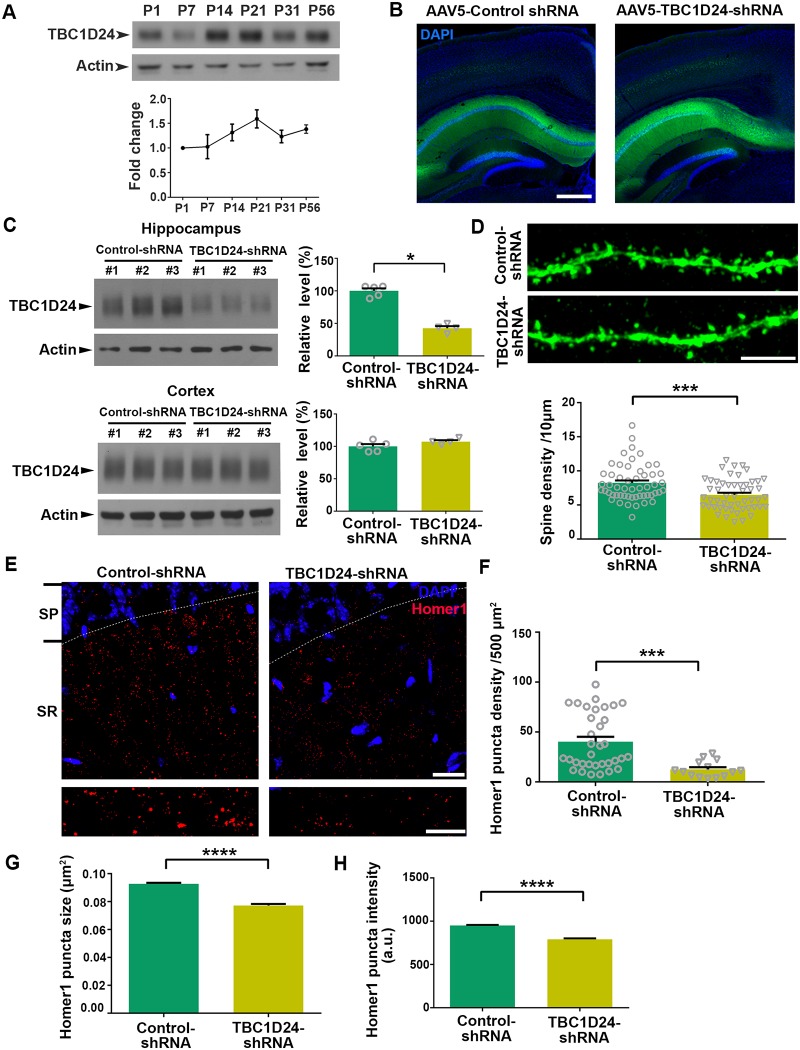
Knockdown of TBC1D24 in adult hippocampus results in reduction of dendritic spine density and affects Homer1 puncta *in vivo*. **(A)** The expression of TBC1D24 increased along postnatal development of the hippocampus. Results were pooled from three independent experiments. **(B)** Representative images of hippocampus injected with AAV5 carrying YFP and the corresponding shRNAs. Scale bar: 500 μm. **(C)** Western blot analysis showing the spatially-restricted knockdown of TBC1D24 after injection of AAV5 carrying control shRNA or TBC1D24-shRNA into the hippocampus. Significant reduction of TBC1D24 expression was observed in the hippocampus one month post-injection with TBC1D24-shRNA, while TBC1D24 expression in the cortex was not affected (n = 4–5 for each condition; mean+SEM; *p<0.05; Mann-Whitney test). **(D)** Representative images of hippocampal CA1 secondary apical dendrites of mice (12 weeks old) after injection of AAV carrying YFP and the corresponding shRNAs. Knockdown of TBC1D24 significantly decreased the number of dendritic spines in hippocampal CA1 neurons (52–53 dendrites from 5 mice were quantified for each condition; mean+SEM; ***p<0.001; Mann-Whitney test). Scale bar: 5 μm. **(E)** Hippocampal slices from mice (12 weeks old) injected with control or TBC1D24-shRNA were immunostained with anti-Homer1 antibody. Representative images of Homer1 puncta in the stratum radiatum (SR) of hippocampus CA1 region. SP = stratum pyramidae. Scale bars: 20 μm (top) and 10 μm (bottom). **(F-H)** The puncta density, size and intensity of Homer1 were significantly decreased in the hippocampus of mice administrated with TBC1D24-shRNA (14–34 images from 5 mice for each condition; mean+SEM; ***p<0.001; Mann-Whitney test in (F); 7119–37789 puncta were analyzed for each condition; mean+SEM; ****p<0.0001; Mann-Whitney test).

Consistent with the up-regulation of TBC1D24 upon maturation of hippocampal neuron *in vitro*, we found that TBC1D24 expression in the hippocampus *in vivo* increased from postnatal two weeks onwards and persisted in adulthood (Fig 4A), indicating that its function extends beyond synapse development at early postnatal stages. To address the potential role of TBC1D24 on dendritic spine maintenance in the adult hippocampus, adeno-associated viruses (AAV) carrying TBC1D24-shRNA or control shRNA were introduced into the hippocampi of adult mice by stereotaxic injection. Expression of the viral particles, which carried the YFP construct, was confirmed to be mostly restricted in the hippocampus (Fig 4B). The viral-mediated delivery of the shRNA was effective and decreased TBC1D24 expression specifically in the hippocampus but not cortex *in vivo* by about 50% (Fig 4C). We then ask whether the *in vivo* knockdown of TBC1D24 in adult hippocampus affects the number of dendritic spines in CA1 neurons. Mice were injected with AAV carrying YFP and control- or TBC1D24-shRNA at low titer in order to visualize isolated dendrites, and knockdown of TBC1D24 significantly reduced the density of dendritic spines (Fig 4D). Knockdown of TBC1D24 in adult hippocampal CA1 neurons also significantly decreased the density, size and intensity of the puncta of Homer1, a major scaffold protein that is commonly used to indicate hippocampal excitatory postsynaptic sites by immunohistochemistry [39, 40] (Fig 4E–4H), further suggesting that TBC1D24 regulates postsynaptic maturation *in vivo*.

### Depletion of TBC1D24 in the adult hippocampus alters animal behaviors and impairs memory formation

**Fig 5:**
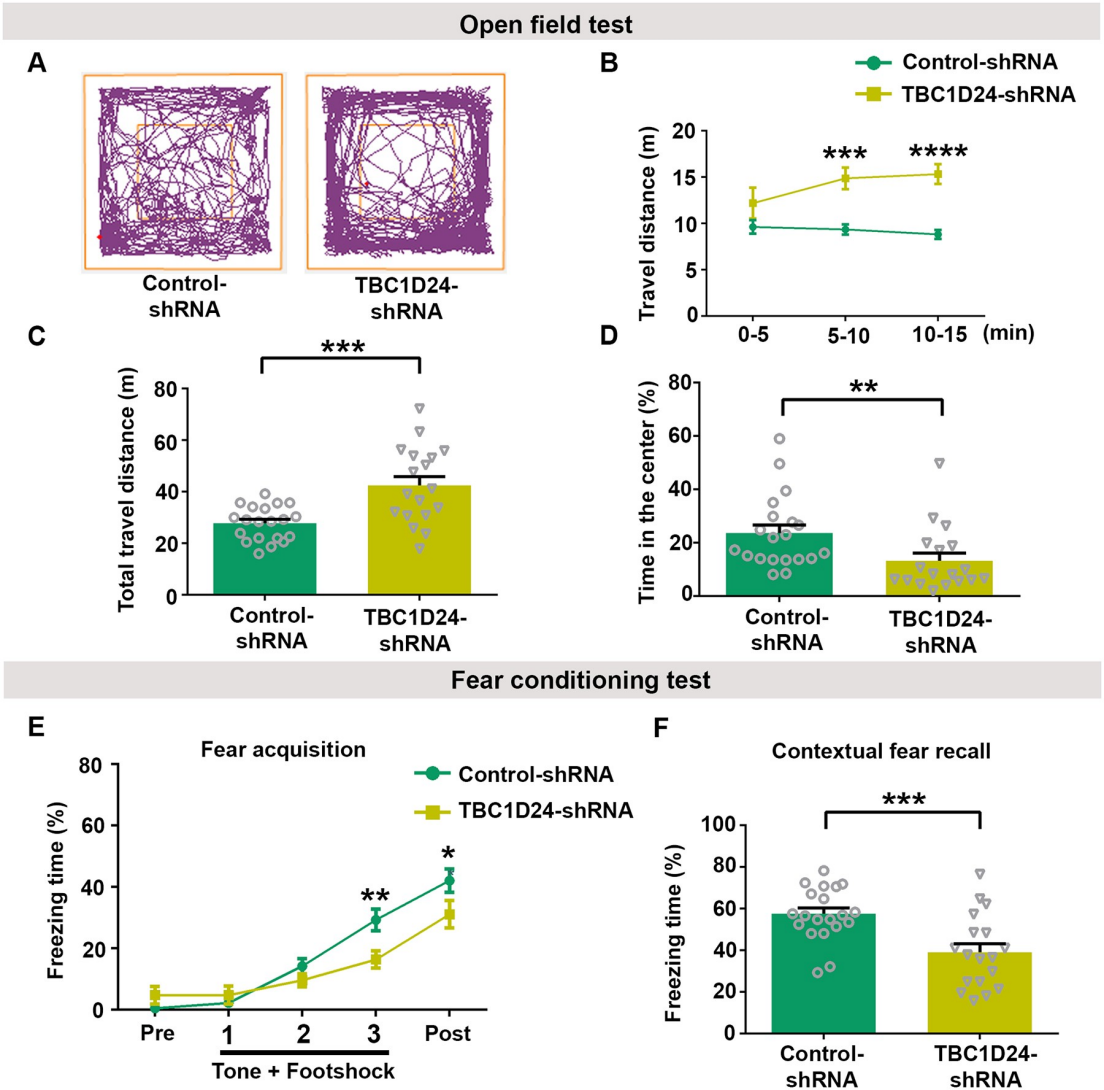
Knockdown of TBC1D24 in adult hippocampus leads to alteration of animal behaviors. **(A)** Representative locomotor activity traces of a control-shRNA-injected mouse and a TBC1D24-shRNA-injected mouse in the open field test. **(B, C)** Mice injected with TBC1D24-shRNA exhibited higher locomotor activity in the open field test during 15-minute observation in comparison with mice injected with control-shRNA. Binned at 5 minutes interval and cumulative data were plotted for travel distance [18–20 mice for each condition; mean+SEM; ***p<0.001, ****p<0.0001; two-way ANOVA followed by Sidak test in (B): delivered gene F (1, 36) = 15.78, p = 0.0003; time F (2, 72) = 2.543, p = 0.0856; interaction F (2, 72) = 5.751, p = 0.0048; ***p<0.001, unpaired Student’s *t*-test in (C)]. **(D)** Mice with knockdown of TBC1D24 manifested increased anxiety, as indicated by less time spent in the center of the open field arena compared to control mice. (18–20 mice for each group; mean+SEM; **p<0.01; Mann-Whitney test). **(E, F)** The average freezing time was measured during habituation for 60 sec (pre), conditioning sessions containing three repeated stimuli (Tone/30 sec + Footshock of 0.5 mA/2 sec) with a 20-sec interval between each stimulus, and end session when mice were left undisturbed in the chamber for 60 sec after stimulus (post). Contextual fear recall tests were conducted at 24 hours after fear conditioning. The freezing time was reduced in mice after TBC1D24 knockdown, indicating that contextual fear learning and memory were impaired [18–20 mice for each group; mean+SEM; *p<0.05, **p<0.01; two-way ANOVA followed by Sidak analysis in (E): delivered gene F (1, 36) = 2.206, p = 0.1462; time F (4, 144) = 84.66, P < 0.0001; interaction F (4, 144) = 6.156, P = 0.0001; and ***p<0.001; unpaired Student’s *t*-test in (F)].

Alteration of dendritic spines in the hippocampus has been linked to abnormal locomotive behavior and impaired spatial memory [41–43]. We therefore determine the consequences of hippocampal TBC1D24 knockdown on these animal behaviors. We performed open field test to determine locomotive behavior of the mice in which hippocampal TBC1D24 level was depleted. The travel distance of the mice injected with TBC1D24-shRNA was significantly higher than those injected with control shRNA (Fig 5A–5C). Mice with TBC1D24 knockdown also showed stronger preference to move at the periphery of the arena, indicating increased anxiety (Fig 5A and 5D). To compare hippocampus-dependent spatial learning and memory between mice injected with control-shRNA and those with TBC1D24 knockdown, contextual fear conditioning was performed. Mice injected with TBC1D24-shRNA showed reduced freezing after the conditioning, indicating impaired fear learning (Fig 5E). Moreover, the impaired learning is associated with reduced freezing 24 hrs after the conditioning in the same environmental context, suggesting defects in the formation of hippocampus-dependent spatial memory (Fig 5F). Taken together, these findings indicate that TBC1D24 plays a crucial role in maintaining dendritic spines in the adult hippocampus *in vivo*, and depletion of this protein beyond synapse formation alters affective behaviors and impairs learning and memory.

### The disease-related F251L substitution destabilizes TBC1D24 protein

**Fig 6:**
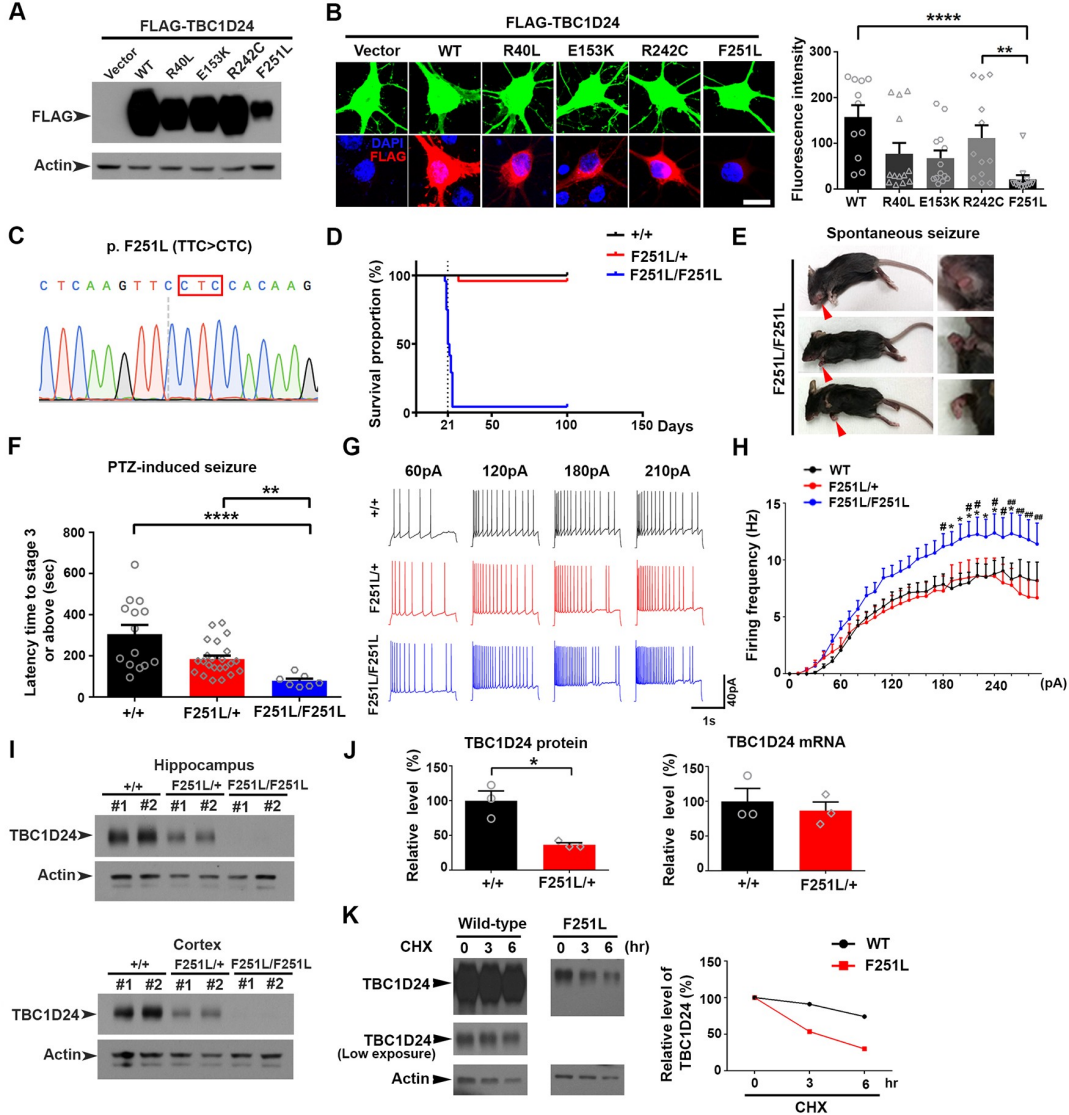
Epilepsy- and ID-associated F251L mutation represses TBC1D24 protein expression and causes spontaneous seizure. **(A)** HEK 293T cells were transfected with wild-type (WT) or four different disease-associated TBC1D24 mutants. Western blot revealed drastic reduction of TBC1D24 protein expression in cells expressing the F251L mutant. **(B)** Hippocampal neurons were transfected with GFP together with FLAG-tagged wild-type or four different disease-associated TBC1D24 mutants, followed by immunostaining with antibodies against GFP and FLAG. Quantification demonstrated lowest expression of the TBC1D24-F251L mutant (11–13 neurons were quantified for each group; mean+SEM; **p<0.01, ****p<0.0001; Kruskal-Wallis test followed by Dunn’s multiple comparisons). Scale bar: 10 μm. **(C)** The nucleotide substitution of the F251L homozygous knock-in mouse was confirmed by Sanger sequencing. **(D)** The cumulative survival rate was plotted against age for wild-type and mutant mice. (n = 8 for wild type; n = 24 for heterozygous TBC1D24^F251L/+^; n = 24 for homozygous TBC1D24^F251L/F251L^ mice). **(E)** Images of carcasses of homozygous TBC1D24^F251L/F251L^ mice that showed signs of maximal seizure (extended tongue and clenched forepaws). **(F)** The homozygous TBC1D24^F251L/F251L^ mice had significantly reduced threshold of PTZ-induced seizure as compared to wild-type and heterozygous mice. Injection of PTZ was performed on mice at postnatal day 18–20 (n = 14 for TBC1D24^+/+^ mice; n = 23 for TBC1D24^F251L/+^ mice; n = 7 for TBC1D24^F251L/F251L^ mice; mean+SEM; **p<0.01; ****p<0.0001;Kruskal-Wallis test followed by Dunn’s multiple comparisons). **(G-H)** Representative traces of action potential from wild-type, heterozygous TBC1D24^F251L/+^, and homozygous TBC1D24^F251L/F251L^ CA1 neurons injected with different currents. The frequency of action potential was significantly increased in CA1 neurons from homozygous TBC1D24^F251L/F251L^ mice (16 cells from 4 wild-type mice; 14 cells from 3 heterozygous TBC1D24^F251L/+^ mice; 16 cells from 4 homozygous TBC1D24^F251L/F251L^ mice; *p<0.05, wild-type vs F251L homozygous; ^#^p<0.05, ^##^p<0.01, F251L heterozygous vs F251L homozygous; two-way ANOVA followed by Sidak analysis; current F (29, 1290) = 31.19; P < 0.0001; genotype F (2, 1290) = 59.35, P < 0.0001; interaction F (58, 1290) = 0.6470, P = 0.9817). **(I)** Western blot analysis showed dosage-dependent reduction of TBC1D24 protein expression in the hippocampus and cortex of heterozygous TBC1D24^F251L/+^ and homozygous TBC1D24^F251L/F251L^ mice (postnatal day 21). **(J)** Real time RT-PCR showed similar expression of *Tbc1d24* mRNA between the hippocampi of wild-type TBC1D24^+/+^ and TBC1D24^F251L/+^ mice (postnatal day 42–44) despite the significant difference in TBC1D24 protein expression. (n = 3 mice for each group; mean+SEM; *p<0.05; unpaired Student’s *t*-test). **(K)** Increased turnover of the F251L mutant of TBC1D24. HEK-293T cells were transfected with wild-type (WT) or TBC1D24 F251L mutant. The cells were then treated with cycloheximide (CHX, 20 μg/ml) for 0, 3, 6 hr, respectively. Presented values were the signal intensities of TBC1D24 in Western blot. Lower exposure for wild-type TBC1D24 was shown in parallel to show its relatively stable expression without saturating the signals.

To better understand the pathophysiological effects of TBC1D24 mutations on synaptic and cognitive functions, we examined a number of individual ID-associated missense mutations which result in the amino acid substitutions R40L, E153K, R242C and F251L [27, 28, 44, 45] (S4 Fig). Remarkably, when expressing the four TBC1D24 mutants in either HEK-293T cells or primary hippocampal neurons, only the F251L substitution specifically led to drastic reduction in protein expression (Fig 6A and 6B).

To confirm the effect of the F251L substitution on TBC1D24 expression and to investigate the consequences of this pathological mutation on brain function, knock-in mice harboring this missense mutation (c.751T>C) were generated (Fig 6C). Both the homozygous and heterozygous F251L knock-in mice could be born, but majority of the homozygous TBC1D24^F251L/F251L^ mice died prematurely around postnatal day 21, while the heterozygous TBC1D24^F251L/+^ mice survived into adulthood (Fig 6D). Despite the early premature death of the homozygous mice, there was no apparent difference in body and brain sizes across the genotypes (S5A Fig). The brain anatomy and cortical migration also appeared grossly normal in the F251L knock-in mice (S5B and S5C Fig). However, the homozygous TBC1D24^F251L/F251L^ mice exhibited spontaneous seizure, as indicated by wild running and convulsion, right before they died (S1 Video). Signs of convulsion such as clenched forepaws and extended tongue [46, 47] were also detected in the carcasses of the homozygous mice (Fig 6E). No such seizure was observed in either the wild-type or heterozygous littermates (n = 3 mice for each genotype). The homozygous TBC1D24^F251L/F251L^ knock-in mice were also more susceptible to seizure induced by pentylentetrazole (PTZ), exhibiting significantly lower latency of seizure induction (Fig 6F). To ask whether the epileptic seizure is associated with increased excitability of hippocampal neurons, we measure the frequency of action potential upon injecting different current steps to CA1 neurons of hippocampal slices from wild-type and knock-in mice. Significantly higher frequencies of action potential were observed in the homozygous TBC1D24^F251L/F251L^ knock-in mice at multiple current steps (Fig 6G and 6H). The homozygous F251L mutation therefore causes increased neuronal excitability and postnatal epilepsy, consistent with the prominent epileptic symptoms observed in patients harboring the same missense mutation [11].

Western blot analysis of cerebral cortices and hippocampi isolated from the heterozygous TBC1D24^F251L/+^ and homozygous TBC1D24^F251L/F251L^ mice confirmed the remarkable reduction of TBC1D24 expression (Fig 6I). Despite the large decrease in TBC1D24 protein level in the hippocampus of heterozygous TBC1D24^F251L/+^ mice as compared to wild-type mice, the level of *Tbc1d24* mRNA was similar between the two genotypes (Fig 6J), indicating that the missense mutation affects TBC1D24 protein expression at the post-transcriptional level. One plausible explanation is increased turnover of the mutated protein. To test this possibility, 293T cells expressing the wild-type or F251L mutant TBC1D24 were treated with the protein synthesis inhibitor cycloheximide to monitor turnover of the exogenously expressed proteins. The F251L amino acid substitution indeed showed enhanced protein turnover (Fig 6K), supporting the notion that the missense mutation disrupts protein stability that leads to significant reduction of TBC1D24 expression in the brains of the knock-in mice.

### Alteration of dendritic spine morphology and excitatory synaptic transmission in homozygous TBC1D24^F251L/F251L^ hippocampal neurons *in vitro*

**Fig 7:**
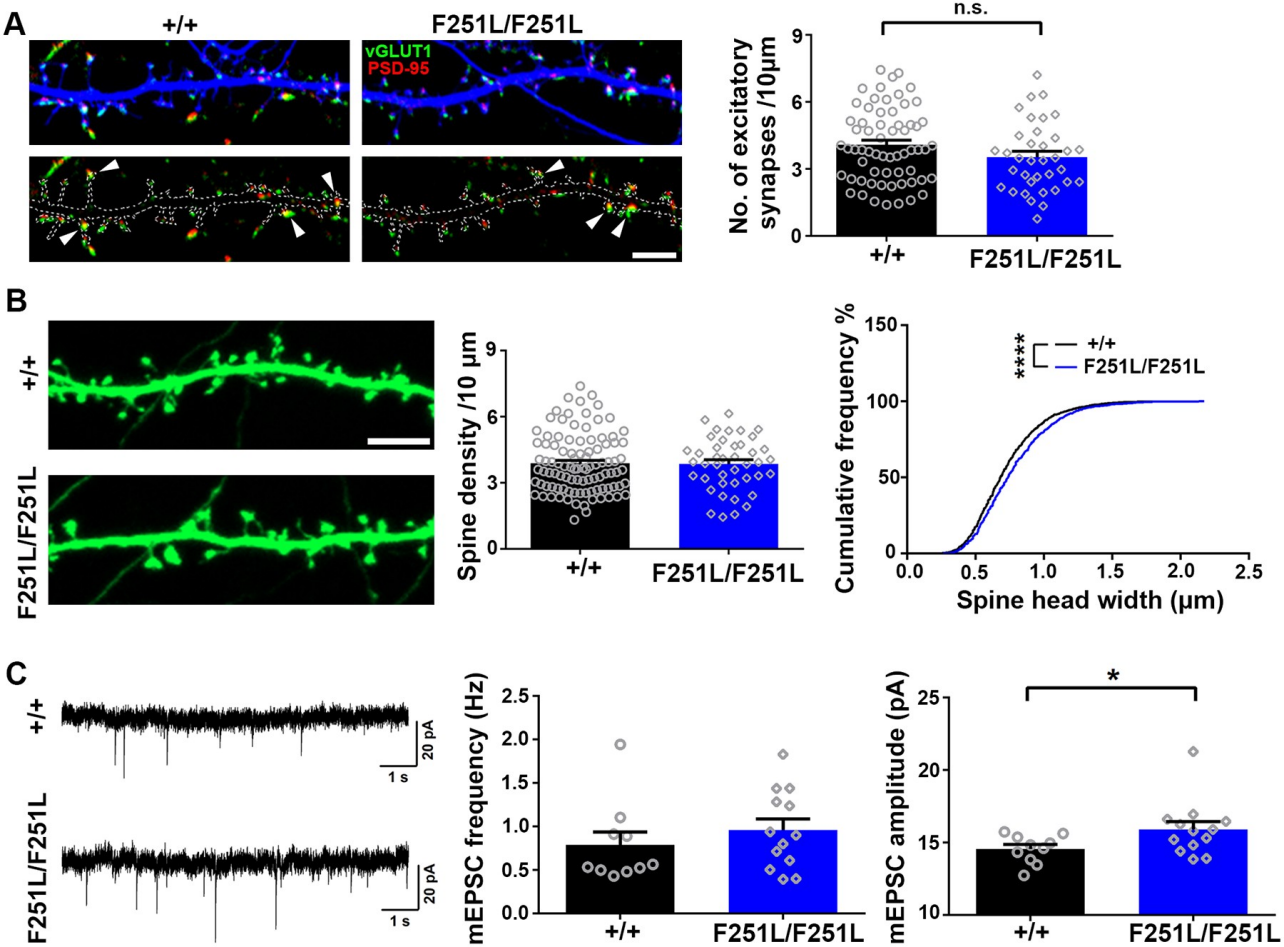
Homozygous TBC1D24^F251L/F251L^ hippocampal neurons show spine enlargement and increased mEPSC amplitude *in vitro*. **(A)** Hippocampal neurons (12 DIV) were transfected with GFP construct and fixed at 9 days post transfection for immunostaining with antibodies against GFP, vGLUT1 and PSD95. The number of excitatory synapses was not significantly different between wild-type and TBC1D24^F251L/F251L^ neurons (36–63 dendrites from two independent experiments for each condition; mean+SEM; p = 0.1133; Mann-Whitney test). Scale bar: 5 μm. **(B)** Hippocampal neurons (12 DIV) were transfected with GFP plasmid, followed by fixation at 6 days post transfection for immunostaining with anti-GFP antibody. The width of spine heads was significantly increased in homozygous TBC1D24^F251L/F251L^ neurons as compared to wild-type control (3233 spines from wild-type and 1375 spines from homozygous TBC1D24^F251L/F251L^ were pooled from three independent experiments; mean+SEM; ****p<0.0001; Mann-Whitney test). There was no significant difference in spine density between wild-type and homozygous knock-in neurons (42–99 dendrites pooled from three independent experiments were quantified for each genotype; mean+SEM). Scale bar: 5 μm. **(C)** Whole cell patch-clamp recording was performed with hippocampal neurons (17–20 DIV) in the presence of TTX and bicuculline. Representative traces were shown. The mEPSC amplitude but not frequency was significantly increased in TBC1D24^F251L/F251L^ neurons (10–13 neurons from three independent experiments for each condition; mean+ SEM; *p<0.05; Mann-Whitney test).

We next dissociated hippocampal neurons from wild-type and the homozygous F251L knock-in mouse embryos to examine the dendritic spines and excitatory synapses after transfection with GFP. The TBC1D24^F251L/F251L^ homozygous neurons showed reduction in synapse density as compared to wild-type control, although the difference did not reach statistical significance (p = 0.1133; Fig 7A). Interestingly, the TBC1D24^F251L/F251L^ neurons possessed significantly larger dendritic spines without change in spine density (Fig 7B). Since larger spine head is correlated with enhanced AMPA receptor current and mEPSC amplitude [48, 49], we also determined the excitatory synaptic transmission of dissociated wild-type and TBC1D24^F251L/F251L^ hippocampal neurons by whole-cell patch recording. The homozygous TBC1D24^F251L/F251L^ neurons showed increased mEPSC amplitudes without change in mEPSC frequency (Fig 7C), which is consistent with their larger dendritic spines. These findings indicate that the early-onset TBC1D24 deficiency due to homozygous F251L mutation alters dendritic spines and excitatory synaptic transmission *in vitro*, but the phenotypes are distinct from TBC1D24 knockdown by shRNA, which depletes TBC1D24 in mature neuron after synapses have been formed.

### Adult heterozygous TBC1D24^F251L/+^ mice display reduced number of dendritic spines *in vivo* and fear memory deficits

**Fig 8:**
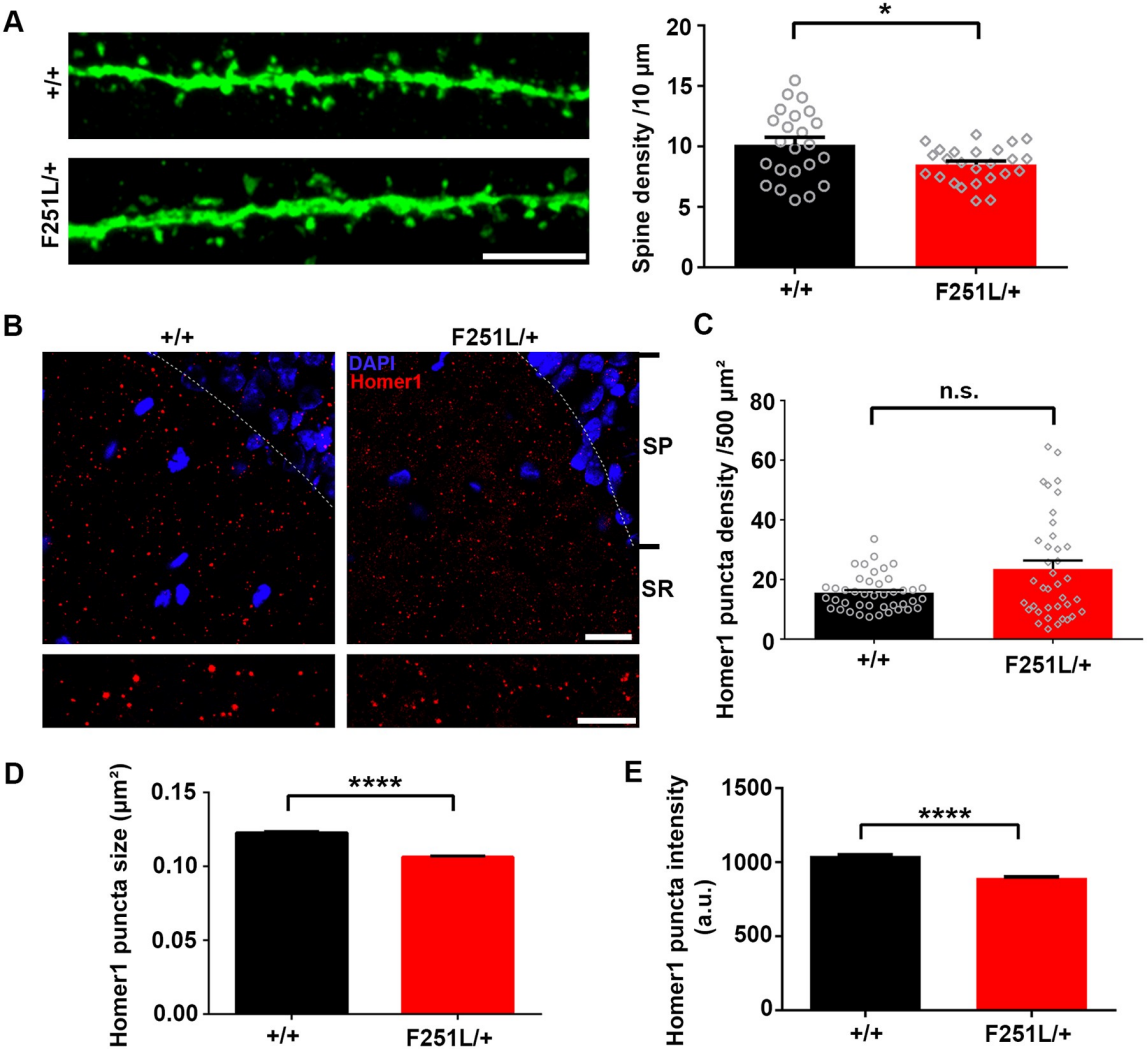
Heterozygous TBC1D24^F251L/+^ knock-in mice exhibit aberrant Homer1 puncta and reduction of dendritic spine density in the hippocampus *in vivo*. **(A)** Representative images of hippocampal CA1 secondary apical dendrites after injection of AAV carrying YFP into the hippocampi of wild-type and heterozygous TBC1D24^F251L/+^ knock-in mice (12 weeks old). Heterozygous TBC1D24^F251L/+^ knock-in mice displayed reduced density of dendritic spines in CA1 of hippocampus (23–25 dendrites from 4 mice were quantified for each genotype; mean+SEM; *p<0.05; upaired Student’s *t*-test). **(B)** Immunohistochemistry of hippocampal slices from wild-type and F251L heterozygous knock-in mice (6 weeks old) with Homer1 staining. Representative images of Homer1 puncta in the stratum radiatum (SR) of hippocampus CA1 region. SP = stratum pyramidae. **(C-E)** The number of Homer1 puncta was not significantly changed (p = 0.1989), while the size and intensity of Homer puncta were significantly reduced in the heterozygous TBC1D24^F251L/+^ knock-in mice [38–42 images from 4 mice were quantified for each genotype; mean+SEM; Mann-Whitney test in (C); 25605–34877 puncta were analyzed for each condition; mean+SEM ****p<0.0001;Mann-Whitney test in (D, E)]. Scale bars: top, 20 μm; bottom, 10 μm.

**Fig 9:**
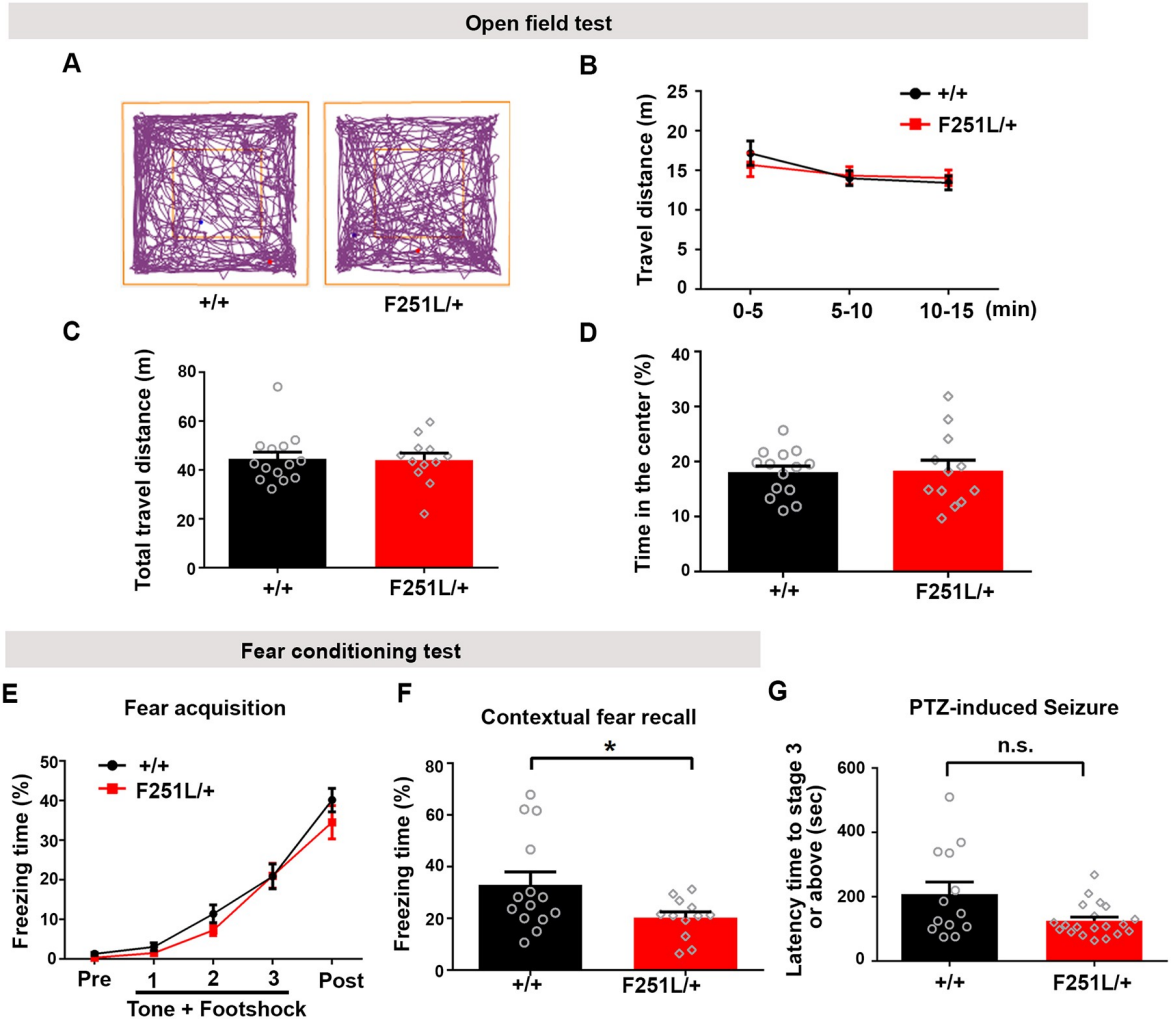
Heterozygous TBC1D24^F251L/+^ knock-in mice display fear memory deficit. **(A)** Representative activity traces from a wild-type mouse and a heterozygous TBC1D24^F251L/+^ mouse in open field test. **(B-D)** Travel distance and the percentage of time in the center of the open field arena were not affected in the heterozygous knock-in mice [(n = 12–14 mice for each genotype; mean+SEM; two-way ANOVA followed by Sidak test in (B): genotype F (1, 24) = 0.01601, p = 0.9004; time F (2, 48) = 5.234, p = 0.0088; interaction F (2, 48) = 0.8021, p = 0.4543]. **(E, F)** The average freezing time was measured during habituation for 60 sec (pre), conditioning sessions containing three repeated stimuli (Tone/30 sec + Footshock of 0.5 mA/2 sec) with a 20-sec interval between each stimulus, and end session when mice were left undisturbed in the chamber for 60 sec after stimulus (post). The heterozygous TBC1D24^F251L/+^ mice showed reduced freezing during contextual fear recall 24hr after fear conditioning (F), but the learning of fear was not affected (E) [n = 12–14 mice for each genotype; mean+SEM; two-way ANOVA followed by Sidak test in (E): genotype F (1, 24) = 2.055, p = 0.1646; time F (4, 96) = 91.72, p < 0.0001; interaction F (4, 96) = 0.5634, p = 0.6898, and *p<0.05, unpaired Student’s *t*-test in (F)]. **(G)** No significant difference in susceptibility to seizure induced by intraperitoneal administration of PTZ between the adult (15–23 weeks) heterozygous TBC1D24^F251L/+^ and wild-type mice (n = 13 for TBC1D24^+/+^ wild-type mice; n = 20 for TBC1D24^F251L/+^ mice; mean+SEM; Mann-Whitney test).

Because majority of the homozygous knock-in mice died at the time of weaning (Fig 6D), we employed the heterozygous TBC1D24^F251L/+^ mice to investigate whether pathological deficiency of TBC1D24 due to gene mutation affects dendritic spines and Homer1 puncta of adult hippocampal neurons *in vivo*, as well as the consequences on animal behaviors. Consistent with the decrease in spine density after *in vivo* TBC1D24 knockdown by shRNA, the hippocampal CA1 neurons of the TBC1D24^F251L/+^ mice possessed fewer dendritic spines (Fig 8A). The size and intensity of Homer1 puncta were also significantly reduced in the stratum radiatum in TBC1D24^F251L/+^ hippocampus, while the number of Homer1 puncta was not changed (Fig 8B–8E). To ask whether the postsynaptic defects in hippocampus alter animal behaviors, the heterozygous TBC1D24^F251L/+^ mice were subjected to a battery of behavioral tasks. Interestingly, despite the strong effect of TBC1D24 knockdown on hyperactivity and anxiety, no significant difference in behaviors was observed between the wild-type and the heterozygous knock-in mice in the open field test (Fig 9A and 9D). However, the heterozygous knock-in mice exhibited reduced fear memory after contextual fear conditioning (Fig 9E and 9F). No significant difference in seizure susceptibility was observed between the adult heterozygous knock-in mice and wild-type control (Fig 9G). Therefore reducing TBC1D24 expression by the heterozygous F251L mutation causes similar defects in dendritic spine maintenance and impaired memory to the acute TBC1D24 knockdown in adult hippocampus *in vivo*.

## Discussion

Increasing number of human genetic studies have unequivocally identified TBC1D24 as an essential protein for normal brain function, but its physiological significance and the pathogenesis of the various disease mutations are far from clear. In addition to the previously characterized presynaptic function, we now present evidence that TBC1D24 is located at the PSD and regulates dendritic spines of the postsynaptic neuron. Using both shRNA-mediated knockdown and generation of transgenic mice, we investigate for the first time how depletion of this protein affects cognitive function and animal behaviors. We further identify the F251L pathogenic mutation disrupts protein stability and drastically reduces TBC1D24 protein expression, and knock-in mice harboring this mutation develop phenotypes that are relevant to the human disorder. In the homozygous mutant mice, substantial loss of TBC1D24 expression during early development leads to increased neuronal excitability, spontaneous seizure and postnatal death, which agrees with the reported cases of epilepsy in most individuals harboring *TBC1D24* gene mutations. On the other hand, the heterozygous F251L mutation does not cause notable seizure but results in dendritic spine defects in the hippocampus *in vivo* that are associated with impaired memory formation at adulthood. Together with the results from shRNA-mediated knockdown of TBC1D24, our study demonstrates a crucial role of TBC1D24 in dendritic spine maintenance of hippocampal neurons *in vivo* that is essential for memory formation. This may explain the occurrence of ID in majority of patients carrying *TBC1D24* loss-of-function mutations.

The current study has unraveled the pathogenesis underlying missense mutation that leads to F251L substitution within the TBC domain. While not constituting part of the cationic pocket, this F251 residue is conserved in TBC1D24 from all species except zebrafish (S6 Fig). Among the four different disease-relevant missense mutations (R40L, E153K, R242C and F251L) in the TBC domain of TBC1D24, only the F251 substitution leads to drastic reduction of TBC1D24 protein expression. At the time when we started to generate *Tbc1d24* knock-in mice to model the disease, there was not yet any published *Tbc1d24* genetic mouse model including the conventional knockout mice. Given that many missense mutations have unidentified significance [50], we chose F251L mutation to develop an animal model for a disease-relevant mutation since it is expected to disrupt TBC1D24 expression like gene knockout, thereby enabling us to understand the physiological function of TBC1D24. Despite the low level of TBC1D24 protein in the F251L knock-in mice, the *Tbc1d24* mRNA expression is comparable with that of the wild-type control, suggesting that this amino acid is important to maintain stability of the protein, which is supported by the cycloheximide-chase assay. This is reminiscent of the ASD-associated missense mutation R87W of neuroligin-4, in which substitution of a single amino acid can destabilize the protein and results in adverse effect on cognitive function [51].

It is noteworthy that there are similarities and differences in phenotypes arising from the TBC1D24 knockdown and knock-in approaches, both *in vitro* and *in vivo*. Either TBC1D24 knockdown by viral delivery of shRNA into adult brain or depletion of TBC1D24 by the F251L heterozygous mutation results in reduction of dendritic spines in adult hippocampal CA1 neurons *in vivo*. Likewise, reduction of spine number is observed in primary hippocampal neurons after TBC1D24 knockdown *in vitro*, which also possess fewer excitatory synapses and smaller mEPSC frequency. However, the homozygous TBC1D24^F251L/F251L^ hippocampal neurons *in vitro* exhibit a very different phenotype: they possess larger spine heads without reduction of spine density, and show elevated synaptic response as indicated by increased mEPSC amplitude without change in frequency. The larger dendritic spines and increased mEPSC amplitude in the TBC1D24^F251L/F251L^ neurons in culture are noteworthy, because neurons deficient in SynGAP also possess larger spine heads and enhanced mEPSCs [52, 53] and both the SynGAP happloinsufficient mice and patients with SynGAP mutations have epilepsy [52, 54]. Given the potential link between spine size and excitability [55] and the spontaneous seizure displayed by the homozygous TBC1D24^F251L/F251L^ mice, the enlarged dendritic spines and enhanced excitatory synaptic response might potentially be linked to increased excitability of the F251L homozygous mice. On the other hand, the number of excitatory synapses and dendritic spines is not significantly affected in homozygous TBC1D24^F251L/F251L^ neurons *in vitro*. There are several possibilities to explain the difference in phenotypes between the TBC1D24 knockdown and F251L homozygous knock-in neurons. First, primary neuron harboring homozygous F251L mutation represents a model of early TBC1D24 deficiency that begins in developing neurons when neurites and synapses are forming; whereas TBC1D24 knockdown represents deficiency in mature neurons since the shRNA was introduced at 15–16 DIV after synapses have already been formed. It appears that the effect of TBC1D24 depletion on dendritic spines and excitatory synapses is stage-specific, similar to what have been described for ARF6 which promotes spine growth in young neurons but disrupts spine maintenance in mature neurons [20]. Therefore, decrease in dendritic spine number may only be observed when TBC1D24 deficiency occurs in mature neurons, such as knockdown beyond synapse formation *in vitro* and *in vivo*, as well as in the adult heterozygous TBC1D24^F251L/+^ mice *in vivo*. Second, besides the difference in time and duration of TBC1D24 depletion, in TBC1D24 knockdown neurons the shRNA was introduced by calcium phosphate precipitation. The reduction of TBC1D24 expression is therefore limited to the sparse GFP-positive postsynaptic neurons and the effect is likely cell-autonomous. In contrast, both the pre- and postsynaptic neurons carrying the F251L mutation exhibit depletion of TBC1D24. It is possible that TBC1D24 deficiency in the surrounding presynaptic terminals exerts an effect on the postsynaptic F251L mutant neuron and may contribute to the difference in spine phenotypes compared to knockdown neuron. Third, embryonic gene deletion may trigger functional compensation, which may underlie the discrepancy in phenotypes caused by genetic knockout and RNAi-mediated knockdown as reported by increasing number of studies [56–58]. Milder phenotype in genetic knockout has also been observed regarding regulators of GTPases. For example, only knockdown but not knockout of the Rap-GEF Epac2 reduces spine density [59, 60], while acute knockdown but not genetic knockout of *Tbc1d24* results in neuronal migration defects [17, 61]. Since TBC1D24 depletion by the homozygous F251L mutation occurs at the very beginning of development, it resembles the deficiency of gene product in knockout animals. We propose that other TBC-domain containing proteins and/or other neuronal ARF-GAPs such as AGAP3, GIT1 and centaurin α1 [22, 62, 63] might compensate for the TBC1D24 deficiency at early stage and help to maintain dendritic spines in the cultured TBC1D24^F251L/F251L^ neurons. Nonetheless, the homozygous TBC1D24^F251L/F251L^ neurons show larger spine heads and increased excitability while the homozygous mutant mice show severe spontaneous seizure, indicating that not all TBC1D24 functions can be compensated.

The *Drosophila* TBC1D24 orthologue Skywalker regulates neurotransmitter release at the presynaptic terminals [15, 16]. The presynaptic function of TBC1D24 has recently been confirmed in mammalian neurons, in which enlarged endosomes are detected in the presynaptic terminals of TBC1D24 heterozygous knockout neurons that are coupled to reduced glutamate release [61]. On the other hand, we found that TBC1D24 is also localized at postsynaptic sites of excitatory synapses. Indeed, data mining reveals that TBC1D24 is among the ~1,500 PSD proteins identified in a proteomic study of human PSD [5]. The TBC domain contains a cationic pocket that binds to phosphoinositides such as PI(4,5)P_2_ and is important to immobilize TBC1D24 at the presynaptic terminals of motor neurons in fly [64]. PI(4,5)P_2_ is also enriched in dendritic spines [65, 66]. It will be interesting to address in the future whether the postsynaptic localization of TBC1D24 also depends on the cationic pocket, and how various *TBC1D24* disease-associated mutations disrupting this binding pocket (R40C, R242C and R293P) affect postsynaptic function. Besides reduction in dendritic spine number, the postsynaptic scaffold protein Homer1 is also affected in the stratum radiatum after depletion of TBC1D24 *in vivo*. Although co-staining with a presynaptic marker has not been performed, given that majority of Homer1 or PSD-95 puncta are co-localized with each other and apposed to presynaptic terminals in the hippocampus *in vivo* [67, 68, 69], the number of postsynaptic puncta after single Homer1 or PSD-95 staining has been used to reflect the density of excitatory synapses [70, 71, 72, 73]. Knockdown of TBC1D24 by shRNA reduces the density of Homer1 puncta in CA1 neurons, while both TBC1D24 knockdown and TBC1D24^F251L/+^ heterozygous mutation lead to reduction of Homer1 puncta size and intensity in the hippocampus *in vivo*. Homer1 puncta can exist in dendritic shaft or outside synapses [74, 75], which may explain why Homer1 puncta density remains unchanged in the TBC1D24^F251L/+^ hippocampus despite reduced spine density *in vivo*. Homer1 is one of the major PSD scaffold proteins that is critical to the structural and functional integrity of dendritic spines [76, 77]. The size and intensity of Homer1 puncta are regulated in response to stimuli that perturb synaptic transmission and plasticity [78], and reduced Homer1 puncta size and intensity are associated with fewer dendritic spines [39, 75]. It is possible that TBC1D24 maintains dendritic spines in part through regulating Homer1 clustering. In this regard, it is noteworthy that Homer1 is present in endosomes and regulates AMPA receptor endocytosis [75, 79]. Given the role of TBC1D24 in regulating endosomal function [61], it will be important to determine whether TBC1D24 directly affects Homer1-mediated endocytosis in dendritic spines in future studies.

Our study indicates that ARF6 is one of the major downstream effectors of TBC1D24 in the maintenance of dendritic spines in hippocampal neuron. ARF6 has a complicated role in dendritic spine morphogenesis: on one hand, ARF6 promotes the formation of dendritic spines in young neuron through endosomal transport of cell adhesion molecules [19, 23]. On the other hand, down-regulation of ARF6 activity is essential for the maintenance of dendritic spines in mature neuron [20], which is mediated in part through a developmental switch of GEF from BRAG1 to BRAG2 [80]. Since TBC1D24 expression is up-regulated along postnatal development and its expression persists in the adult hippocampus, we propose that TBC1D24 is one of the major regulatory proteins that keeps ARF6 activity under control in order to maintain the proper number of dendritic spines in the mature brain. It should be noted that expression of dominant-negative ARF6 partially but not fully reverses the reduction of spine number induced by TBC1D24-shRNA. This agrees with a very recent study showing that dominant-negative ARF6 only partially rescues the impaired axonal growth after TBC1D24 knockdown in young neuron [35]. It is therefore likely that TBC1D24 mediates its action in neuron partly through suppression of ARF6 activity, but there are other parallel signaling pathways involved. Besides expression of dominant-negative ARF6, we also demonstrate that the Brefeldin-resistant cytohesin inhibitor secinH3 can partially reverse the decrease in spine density induced by TBC1D24 knockdown. SecinH3 can inhibit ARF6 [18, 38] since the ARF6-GEFs ARNO and ARNO2 belong to the cytohesin family and are expressed in hippocampal neuron [81,82]. However, secinH3 does not specifically inhibit ARF6, and the combined effect of not just ARF6 inhibition but also other ARFs might explain why secinH3 treatment alone reduces synapse density while expression of dominant-negative ARF6 does not. We therefore do not rule out the possibility that other ARFs besides ARF6 are also downstream targets of TBC1D24 in the maintenance of dendritic spines. The possibility of Rab-GTPases as downstream targets of TBC1D24 should also be considered. The *Drosophila* TBC1D24 ortholog Skywalker regulates presynaptic release at the neuromuscular junction through suppressing the activity of Rab35 [16], and most TBC domain-containing proteins are Rab-specific GAPs. TBC1D24 is one of the few members of the TBC domain-containing protein family in which the arginine finger is absent, and other TBC proteins that lack arginine finger (USP6 and TBC1D3) do not interact or regulate Rab-GTPases [83, 84]. Although we also show that the mammalian TBC1D24 does not interact with Rab35 in the co-immunoprecipitation experiment, it should be noted that GAPs and GEFs can influence small GTPase activity without stable interaction at basal conditions [85, 86]. There are about 60 mammalian Rab-GTPases and only few of them have been examined for activity regulation by the arginine finger-lacking TBC domain-containing proteins including TBC1D24. Future comprehensive study on the activity of individual Rab-GTPases will be required to determine if any specific Rab-GTPases directly act downstream of TBC1D24 in mammalian neurons.

Neurodegeneration is observed in lethal epileptic encephalopathy caused by TBC1D24 truncating mutation as well as in flies lacking Skywalker [15, 87]. Despite early lethality at 3 weeks after birth, neuronal loss is not apparent in the forebrains of the TBC1D24^F251L/F251L^ homozygous mice. However, the F251L knock-in mice display severe seizure at around postnatal day 21 that leads to lethal convulsion. While this manuscript was under preparation, a very recent study reported generation of TBC1D24 knock-in mice that carry non-sense mutation leading to truncation of the protein [88]. Similar to the TBC1D24^F251L/251L^ mice in our study, the homozygous mice carrying non-sense TBC1D24 mutation also undergo early death at three weeks after birth due to spontaneous seizure [88]. The F251L knock-in mice in our study and transgenic mice harboring non-sense mutation reported by Tona et al. [88] will be valuable for future elucidation of the mechanistic link between TBC1D24 deficiency and epilepsy.

Given the strong association of *TBC1D24* mutations with ID, it is important to characterize how TBC1D24 deficiency *in vivo* affects animal behaviors including learning and memory. Despite the emergence of TBC1D24 transgenic mouse models recently [61,88], the behavioral changes associated with TBC1D24 gene mutations have not been studied, probably because of early lethality of the mice. The TBC1D24^F251L/F251L^ mice in our current study also suffer early death around three weeks after birth. We therefore turn to heterozygous knock-in mice to examine the effect of TBC1D24 depletion on cognitive function, and reveals that TBC1D24 is required for hippocampus-dependent contextual fear memory formation. The memory deficits corroborate with impaired postsynaptic maintenance in the hippocampal CA1 neurons, in which smaller Homer puncta and fewer dendritic spines were detected in the TBC1D24^F251L/+^ heterozygous mice. Although many *TBC1D24* mutations identified in patients are either compound heterozygous or homozygous, individuals with heterozygous non-sense mutation and hence TBC1D24 haploinsufficiency have been reported to show seizure and intellectual disability [44,89], indicating that the proper expression level of TBC1D24 is crucial for cognitive function. The causative link between hippocampal TBC1D24 deficiency and impaired contextual fear memory is verified by mice with hippocampal injection of TBC1D24-shRNA. Interestingly, compared to the heterozygous F251L mice, acute knockdown of hippocampal TBC1D24 causes additional behavioral alterations including hyperactivity and increased anxiety, and they also display more severe synaptic defects by having significant reduction of Homer1 puncta density besides puncta size and intensity. Mice with adult-onset TBC1D24 depletion through shRNA therefore generally exhibit more severe phenotypes than the heterozygous F251L mice in which TBC1D24 deficiency begins during development. This is consistent with the emerging studies reporting more severe phenotypes caused by RNAi-mediated knockdown than genetic knockout, and can potentially be explained by functional compensation in response to the embryonic gene depletion [56–58]. Interestingly hyperactivity is also observed in flies harboring *sky* mutation [64]. Previous characterization on patients with *TBC1D24* gene mutations mostly focuses on seizure and intellectual disability. Our findings suggest that anxiety and hyperactivity are also features that warrant further investigation.

Taken together, our study provides new insights onto the subcellular localization and function of TBC1D24 in neuron, and identifies impaired dendritic spine maintenance as a plausible key pathogenic mechanism that contributes to cognitive impairment caused by *TBC1D24* gene mutations. Although many patients carrying *TBC1D24* gene mutations show early-onset epilepsy and developmental delay [11], our findings on mice with acute hippocampal TBC1D24 knockdown *in vivo* indicate that adult-onset depletion of TBC1D24 can produce detrimental effect on cognitive function and behaviors. This raises the interesting possibility that therapeutic intervention at the adult stage may still have beneficial effect on patients with *TBC1D24* mutations. Our findings on the reversal of spine defects of TBC1D24-depleted neurons by SecinH3 also raise the possibility of cytohesin inhibition as a potential therapeutic strategy for the increasing number of patients carrying *TBC1D24* gene mutations.

## Materials and methods

### Ethics statement

All animal experiments were approved by the committee on the use of live animals in teaching and research at the University of Hong Kong.

### Animals

Transgenic C57/BL6J mouse model harboring TBC1D24-F251L mutation was generated by Biocytogen using CRISPR/Cas9 system. Two target sequences CCA TGATAGAGTCAGGCTTTCCG and AGGAAGCTGGGTATTACCAG GGG (PAM sequence underlined) were used to design guide RNA (sgRNA). The Cas9/sgRNA constructs together with targeting knock-in expressing vector were microinjected into the pronuclei of fertilized mouse eggs. Newborn mice were screened by Sanger sequencing for successful substitution of F251L. We bred heterozygous mice for the generation of wild type and homozygous mice. Other animals were obtained from laboratory animal unit at the University of Hong Kong.

### Antibodies, chemicals and DNA constructs

The following primary antibodies were commercially available: TBC1D24 (Aviva), MAP2 (Sigma and abcam), tdTomato (Rockland), Bassoon (abcam), PSD-95 (Thermo Fisher Scientific and NeuroMAB), vGLUT1 (Sigma and Synaptic Systems), Homer 1 (Synaptic Systems), GAD-67 (Millipore), GFP (Invitrogen and Aves Lab), FLAG and actin (Sigma), Brn2 (GeneTex) and Tbr1 (abcam) and NeuN (Cell Signaling). Secondary antibodies were Alexa-conjugated antibodies (Invitrogen) and horseradish peroxidase-conjugated goat anti-rabbit IgG or anti-mouse IgG (Cell Signaling). SecinH3 was purchased from TOCRIS, and tetrodotoxin (TTX) was from abcam. The pCAG_PSD95. FingR-eGFP-CCR5TC plasmid was from Don Arnold (Addgene plasmid #46295). The Myc-Rab35 WT, S22N and Q67L constructs were from Peter McPherson (Addgene plasmid # 47433; # 47435; # 47434).

Two shRNAs targeting rat TBC1D24 were created by inserting a 19nt (5’-GGGTATTACTGTCAAACAG-3’, and 5’-GGAGATGAGAGACATTTGG-3’) into BglII and XhoI restriction sites of pSUPER vector (Oligoengine). For the generation of control- or TBC1D24-shRNA in single plasmid containing GFP-coding sequence, the shRNA sequence was digested from pSUPER backbone and subcloned into pSUPER.neo+GFP vector (a gift from Dr. Dan Ohtan Wang) using EcoRI and KpnI restriction sites. The human TBC1D24 expression construct was generated by subcloning the human TBC1D24 clone (Kazusa DNA Research Institute) into EcoRI and XhoI restriction sites of pcDNA3 vector using primers with FLAG at the N-terminal. Wild-type TBC1D24 resistant to the shRNA was created by site-directed mutagenesis using full-length human TBC1D24 as template. The primers used were as follows: RNAir-TBC1D24 forward 5’- CGTCAGCGTGAGGGAAATGCGAGATATCTGGTCCTGGGTC -3’; reverse 5’-GACCCAGGACCAGATATCTCGCATTTCCCTCACGCTGACG -3’.

In order to have the same wild-type DNA sequence of TBC1D24 (transcript variant 1) as stated in NCBI database, a site mutagenesis (p.L295F) was made by using the primers: forward 5’-TCGCCATCCGCCTCTTCTCCCGCAAG -3’; reverse 5’- CTTGCGGGAGAAGAGGCGGATGGCGA-3’. Wild type and mutated (T27N and T157A) human ARF6 constructs were gifts from Peter Peters (Maastricht University) and Eunjoon Kim (KAIST, Korea). The ARF6 inserts were amplified from plasmids and subcloned into KpnI and XhoI restriction sites of pcDNA3 vector. The primers used were as follows: forward 5’- CGGCCAGGTACCGCCACCATGGGGAAGGTGCTATCC- 3’; Reverse 5’- GTTGCACTCGAGCTATTAAGCGTAATCTGGAAC- 3’.

The expression constructs of different TBC1D24 variants and ARF6-Q67Lwere generated by site-directed mutagenesis using RNAir-TBC1D24 (transcript variant 1) and human ARF6 as template. Primers designed by QuikChange were as follows: TBC1D24-R40L: forward: 5’-AGCAGCTGGCGCTCCAGGGCTACTG -3’; reverse:5’- CAGTAGCCCTGGAGCGCCAGCTGCT—3’; TBC1D24-E153K: forward:5’-GGCCGAGTGCTTCAAGAAGGCCTGCCG-3’;reverse: 5’- CGGCAGGCCTTCTTGAAGCACTCGGCC- 3’; TBC1D24-R242C, forward: 5’-CAAGGTGCTGTACTGCGTGGCGCTGGC- 3’; reverse: 5’- GCCAGCGCCACGCAGTACAGCACCTTG -3’.TBC1D24-F251L: forward: 5’-GGCCATCCTCAAGTTCCTCCATAAGGTGAGGGC—3’; reverse: 5’- GCCCTCACCTTATGGAGGAACTTGAGGATGGCC—3’. ARF6-Q67L: forward 5’-GATGTGGGCGGCCTGGACAAGATCCGG- 3’; reverse 5’-CCGGATCTTGTCCAGGCCGCCCACATC- 3’. For mutagenesis, the PCR was performed with high-fidelity *Pfu* DNA polymerase (Agilent Technologies). The PCR products were digested by DpnI at 37 °C for 2–3 hours and then transformed into E.coli. For generating recombinant AAV vector that carried shRNA targeting mouse TBC1D24, the same region as targeted by rat TBC1D24 shRNA was used to construct the shRNA that targeted mouse TBC1D24. The control shRNA or mouse TBC1D24 shRNA together with H1 promoter in pSUPER was amplified and subcloned into EcoRI and HindIII sites of pAAV-CaMKIIα-eYFP (Neurosciences Institute of Stanford University). The primers used were as follows: T7 promoter forward primer 5′-TAATACGACTCACTATAGGG-3′ and control shRNA reverse: 5′-GTTGCAAAGCTTTTCCAAAAAGGCTACCTCCATTTAG-3′ or mouse TBC1D24 shRNA reverse 5′-GTTGCAAAGCTTTTCCAAAAAGGGTATAACTG-3′. The recombinant AAV construct was packaged into AAV5 capsid particles by UNC Vector Core. Sterile artificial cerebrospinal fluid was used to dilute AAV5 virus for injection. All constructs were verified to be correct by Sanger sequencing.

### Primary cell culture and transfection

Primary hippocampal neurons and cortical neurons were prepared from day 18–19 embryos of Sprague Dawley rats or day 16–18 embryos of C57BL/6J mice as previously described [90]. Hippocampal neurons were cultured on 18-mm coverslips or 35-mm dishes coated with 1mg/ml of poly-D-lysine (Sigma, P0899) at high density (1.4 x 10^5^ cells per coverslip for dendritic spine analysis and mEPSC recording, or 4–5 x 10^5^ cells per 35-mm dish for biochemical analysis) or low density (0.4 x 10^5^ cells per coverslip for vGLUT1/PSD-95 immunocytochemistry and in situ hybridization) in Neurobasal medium supplemented with 2% B27 and 0.25% L-glutamine (Invitrogen). For Western blot analysis, cortical neurons were plated on 35 mm dishes or 60 mm dishes (1x 10^6^ cells per 35mm dish; and 2 cortices per 60 mm dishes) coated with 0.1mg/ml of poly-D-lysine and cultured with Neurobasal medium supplemented with 2% B27, 0.5% L-glutamine and 10 mM D-glucose. For culturing hippocampal neurons from knock-in mouse embryos, three-quarters neurons from each embryo were plated onto one coverslip to prepare high-density neuronal culture for dendritic spine analysis and mEPSC recording; and one-quarter neurons onto one coverslip to prepare low-density neuronal culture for vGLUT1/PSD-95 immunocytochemistry, respectively.

Two transfection methods were employed to introduce plasmids into cultured primary neurons. To test knockdown efficiency of shRNA in primary neurons by Western blot, cortical neurons were used since much more proteins can be extracted from cultured cortical neurons than hippocampal neurons. Nucleofection which has relatively high transfection efficiency (~30%) was employed to transfect cortical neurons in order to observe the knockdown effect. Electroporation of control shRNA or TBC1D24 shRNA into cortical neurons was carried out using the Neon nucleofector transfection system. A total of 1x10^6^ suspension cells were electroporated in each reaction with the parameters of 1600v pulse voltage and 20 ms pulse width. After electroporation, cells were plated on 35mm dish and cultured for 5 days before Western blot analysis. In contrast, calcium phosphate was used to transfect hippocampal neurons because the method typically results in less than 1% neurons being transfected, which is advantageous because it allows the labelling of isolated dendrites by GFP that is critical to the analysis of individual dendritic spines. Cells were transfected with different plasmids using calcium phosphate precipitation according to previous description [31]. For co-transfection of expression construct or shRNA with GFP, the construct of interest was in four-fold excess with that of GFP in the transfection mix, and we have found that all the neurons that express the less abundant construct in the transfection mix also express the other construct. Therefore GFP expression should be a reliable surrogate for shRNA or cDNA expression in the co-transfection experiments.

### In situ hybridization

To visualize subcellular localization of *Tbc1d24* mRNA in hippocampal neurons, RNA *in situ* hybridization was performed using ViewRNA ISH Cell Assay kit (affymetrix) as previously described [31]. The sense and antisense probes were designed against rat TBC1D24 (NM_001105769) by affymetrix.

### Real-time qPCR

For each wild-type or mutant mouse brain (postnatal day 44), one hippocampus was lysed by Trizol (Invitrogen) and then subjected to RNA extraction using RNeasy Mini Kit (Qiagen), while the other one was subjected to protein extraction and Western blot analysis. Total RNA was transcribed into cDNA by RT-Superscript kit (Invitrogen), and the cDNA was used as template for qPCR (performed in triplicate) using TB Green Premix Ex Taq (Takara) and Roche LightCycler480 II Real-time PCR. Relative mRNA expression was quantified using Second Derivative Maximum Method in the LightCycler 480 Software developed by the manufacturer.

### Co-immunoprecipitation and cycloheximide treatment

To overexpress wild-type and TBC1D24 mutants, HEK-293T cells cultured on 100 mm dishes with 80% confluence were transfected with various plasmids using Lipofectamine 2000 (Thermo Fisher Scientific) according to manufacturer’s instruction. Cell lysate was collected 24 hours after transfection for Western blot analysis. For co-immunoprecipitation, HEK293T cells were co-transfected with FLAG-tagged wild-type TBC1D24 and HA-tagged wild-type, T27N, Q67L ARF6 mutations or Myc-tagged wild-type, S22N, Q67L Rab35 mutations. Cells were lysed at 36 hr post transfection with cold lysis buffer (50 mM Tris, pH 7.5, 150 mM NaCl, 0.1% sodium dodecyl sulfate, 1% Nonidet P40) supplemented with protease inhibitors as previously described [13]. The cell lysate was gently rocked in cold room for 60 min and then cleared by centrifugation at 13000 rpm for 10 min at 4 °C. For immunoprecipitation of FLAG-tagged TBC1D24, one mg of cell lysate from each condition were rocked gently and incubated with ANTI-FLAG M2 Affinity Gel (Sigma) at 4 °C for 2 hr (15 μl of beads slurry for each reaction). Beads were pelleted by centrifugation at 3000 x g for 1 minute and washed 3 times with 0.5 mL lysis buffer each time. After the last wash, protein and any potential binding partners were eluted with 40 μl of 2x sample buffer (5X sample buffer: 300 mM Tris-HCl buffer pH 6.8, 10% (w/v) SDS, 25% (v/v) beta-mercaptoethanol, 50% (v/v) glycerol, 0.05% (w/v) bromophenol blue) by boiling for 6 min. To investigate the protein turnover of TBC1D24, HEK 293T cells were transfected with TBC1D24 wild-type or F251L mutants. 24 hr post transfection, cells were treated with 20μg/mL cycloheximide for 0, 3, 6 hr. Cell lysate was collected at the different time points for Western blot analysis.

### Neuronal cell lysate and PSD fractionation for Western blot analysis

To examine the expression of TBC1D24, hippocampal neurons, cortical neurons, HEK-293T cells, or brain tissues were lysed by RIPA containing various protease and phosphatase inhibitors (10 μg/ml soybean trypsin inhibitor, 10 μg/ml, leupeptin, 10 μg/ml aprotinin, 2 μg/ml antipain, 30 nM okadaic acid, 5 mM benzamidine, 1 mM sodium orthovanadate, 1 mM PMSF, 1 mM sodium fluoride, 100 mM beta-glycerophosphate). The lysate was gently rocked in cold room for 45 min and then cleared by centrifugation at 13000 rpm for 10 min at 4 °C.

The synaptosome and PSD fractions were prepared as previously described [91]. In brief, mouse brains were homogenized in HEPES buffer (0.32 M sucrose, 4 mM HEPES [pH 7.4]). The homogenate (Hom) was centrifuged at 900 ×g at 4 °C for 10 min to remove the pelleted nuclear fraction (P1), and the supernatant (S1) was centrifuged at 10,000 × g for 15 min to yield crude synaptosomal fraction (P2) and cytosol/light membranes (S2). The washed P2 fraction (P2′) was subjected to hypoosmotic shock and lysis before centrifugation at 25,000 x g for 20 min to obtain cytoplasm of lysed crude synaptic vesicle (S3) and synaptosome (P3) fraction. P3 fraction was resuspended and centrifuged in a sucrose gradient (0.8M, 1.0M and 1.2M sucrose) to yield the synaptic plasma member (SPM) fraction. SPM was collected at the interface between 1.0 M and 1.2 M sucrose. The PSD fraction was extracted from the SPM fraction by lysis with 0.5% Triton X-100 for 15 min and centrifuged at 32,000 × g for 20 min. The supernatant was Triton X-100 soluble fraction (TS) which contained presynaptic membranes.

Cell lysate and different brain fractions were boiled in sample buffer, separated by SDS-PAGE, and transferred onto PVDF membranes, followed by incubation with primary antibody and HRP-conjugated secondary antibody. The HRP signal was detected by ECL (Thermo Scientific) and quantified by densitometry using Photoshop software.

### Electrophysiology

Whole cell recordings were obtained from hippocampal neurons at 16–19 DIV using the MultiClamp 700B amplifier (Molecular Devices). For miniature excitatory postsynaptic currents (mEPSCs) recording, tetrodotoxin (TTX, 1 μM) and bicuculline (20 μM) were added into the external solution to block action potentials and the inhibitory current from GABA receptor. The pipettes had a resistance of 3–5 MΩ with the internal solution consisting of (in mM): 115 CsCl, 10 HEPES, 2 MgCl_2_∙6H_2_O, 4 NaATP, 0.4 NaGTP, 0.5 EGTA, and pH was adjusted to 7.2–7.4 by CsOH. Neurons were perfused with the external solution of the following composition (in mM): 110 NaCl, 5 KCl, 2 CaCl_2_, 0.8 MgCl_2_, 10 HEPES, 10 Glucose, and pH was adjusted to 7.2–7.4 by NaOH. The signals were filtered at 2 kHz and sampled at 20 kHz using the Digidata 1440A (Molecular Devices). The holding potential was at -70 mV, and only those recordings that lasted for 5 to 10 minutes were used for analysis by MiniAnalysis (Synaptosoft).

For the investigation of excitability of CA1 neurons, whole cell patch-clamp recording was conducted in dorsal hippocampal CA1 brain slices from postnatal day 17–20 mice as previously described [92]. Mice were perfused by ice-cold high sucrose dissection buffer (HSDB) after euthanized. The brains were taken out immediately and submerged in pre-cold HSDB. Coronal brain slices containing CA1 were sectioned at interval of 250 μm by vibrotome. Slices were recovered in warm artificial cerebral spinal fluid (aCSF) at 32°C for 15 minutes, followed by incubation at room temperature. The recordings were performed in aCSF at room temperature. HSDB consisted of the following (in mM): 87 NaCl, 75 sucrose, 2.5 KCl, 1.2 NaH_2_PO_4_, 30 NaHCO_3_, 25 glucose, 20 HEPES, 5 Na-ascorbate, 3 Na-pyruvate, 2 thiourea, 10 MgSO_4_ and 0.5 CaCl_2_. aCSF consisted of the following (in mM): 119 NaCl, 2.5 KCl, 1 MgCl_2_, 2 CaCl_2_, 26 NaHCO_3_, 1.23 NaH_2_PO_4_ and 10 glucose. All solutions were oxygenated by 95% O2 and 5% CO2. Internal solution consisted of the following (in mM) 131 K-gluconate, 20 KCl, 8 NaCl, 10 HEPES, 2 EGTA, 2 NaATP and 0.3 NaGTP, pH 7.3, osmolarity 290 mOsm/L. The glass micropipette was filled with internal solution (resistance 4–6 MΩ) and connected to the electrode for recording. The excitability of CA1 pyramidal neurons was examined by measuring the firing frequency during step-current injection in current-clamp mode. 30 current steps (from 0 to 290 pA, with 10 pA increment of each step current injection, 2-s duration of stimulation, 10-s interval) were applied to neurons. The frequency of action potential of each current step was measured.

### Stereotaxic injection and preparation of hippocampal slices

Each mouse (aged at 8 weeks) was anaesthetized by intraperitoneal injection of a ketamine/xylazine cocktail (120 mg/kg and 18 mg/kg) and mounted on animal stereotaxic instrument (Stoelting). Injection of AAV5 virus was performed using a WPI NanoFil syringe with a 33-gauge needle connected to an infusion pump (KD Scientific). A small volume of viral solution (500 nl) was injected bilaterally into the hippocampus (2.1 mm posterior to bregma point, 1.70 mm lateral to midline and 1.6 mm deep) at an infusion rate of 100 nl/min. The needle was kept in place for 5 minutes prior to withdrawal for complete diffusion of virus solution. Following AAVs administration, mice were placed individually into intensive care unit warmed to approximately 35°C until complete recovery. The mice were then administrated subcutaneously with buprenorphine (Temgesic) at a dosage of 0.1 mg/kg for 6 times at 12 hours interval. At four weeks post stereotactic injection, mice were deeply anesthetized with ketamine/xylazine and perfused transcardially with D-PBS, followed by 4% paraformaldehyde (PFA). The brains were then removed and submerged in 4% PFA at 4 °C for post fixation. The post-fixed brains were sectioned coronally at 50 μm thickness using vibrating blade microtome (Leica VT1000S) for immunohistochemical staining and microscopic imaging.

### Immunohistochemistry

Anesthetized mice were perfused with cold PBS. After perfusion, the brains were dissected, immersed in 4% PFA at 4 °C overnight, then cryoprotected in graded sucrose solution at 4 °C: 10% for 2 hours, 20% overnight and finally 30% sucrose for another overnight. Brains were embedded in OCT. After sectioning at 10 μm and air dried, samples were used immediately for immunohistochemistry or stored at -80 °C. Slices were air dried and rinsed in PBS. Antigen retrieval was achieved by immersing the slices in pre-warmed citrate buffer (PH = 6.0) at 95°C for 10 min. Sections were blocked by 10% goat serum diluted in PBS with 0.3% Triton-X100 for 1 hour, and then incubated with primary antibodies diluted by blocking solution at 4 °C overnight. On the next day, sections were incubated with fluorescent-secondary antibodies at room temperature for 2 hours and then counterstained with DAPI for 15 minutes. After carefully rinsed with PBS, sections were mounted with hydromount medium (National Diagnostics, USA).

For the analysis of dendritic spines, brain sections were prepared by vibrating blade microtome. Brain slices were blocked with blocking buffer (10% goat serum (Gibco) and 0.3% Triton X-100 in D-PBS) at room temperature for 1 hour on shaker, followed by incubation with primary antibody against GFP diluted in blocking buffer at 4°C for two overnights on shaker. Alexa Fluor-conjugated secondary antibodies (Invitrogen) and DAPI (Thermo Fisher Scientific) were added to incubate the slices for 2 hours at room temperature. The slices were then mounted on slides and visualized with a confocal microscope.

### Immunocytochemistry, image acquisition, and quantitative analysis

Cells were incubated with blocking buffer [0.4% Triton X-100 (v/v) and 1% BSA (w/v)] for 45 min at room temperature and incubated with primary antibody in blocking buffer at 4 °C overnight. Cells were washed 3 times with washing buffer (0.02% Triton X-100 and 0.25% BSA in D-PBS), and incubated with Alexa-conjugated secondary antibody (1:1000 diluted in 0.02% Triton X-100 and 1% BSA in PBS) at room temperature for 1 hour. The cells were washed twice with washing buffer and once by D-PBS, and mounted with hydromount medium (National Diagnostics). To visualize the morphology of GFP-transfected neurons, cells were incubated with GFP antibody (1:2000) in GDB buffer (0.2% gelatin, 0.6% Triton X100, 0.9M NaCl, 33mM phosphate at pH 7.4) at 4 °C overnight, and washed three times with washing buffer (0.5 M NaCl, and 20mM phosphate with pH7.4). Cells were incubated with Alexa 488-conjugated secondary antibodies (1:2000 diluted in GDB buffer) at room temperature for 1 hour, then washed three times by the washing buffer, and mounted with hydromount medium.

Olympus Fluoview confocal and Carl Zeiss LSM 700, 780 and 800 laser scanning microscopes were used to acquire fluorescent images at a resolution of 1024 x 1024 pixels with the following parameters: oil-immersion 40x (NA_1.40) or 63x (NA_1.40) objectives, a pinhole of 1 AU for each channel, 1 or 1.3–1.6 optical zoom, averaged 2 times, scan speed 6–8, 0.35–0.60 μm interval with 8 or 16-bit dynamic range. For super-resolution imaging, Zeiss Elyra S1 SIM installed with Zen 2.3 imaging software was used to take images with the following settings: 1024 x 1024 pixels frame size, 63x oil-immersion objective (NA_1.40), 3 rotations grating, 5 phases, 1.0 optical zoom, averaging 1 time, with optimal Z interval. For Airyscan imaging, LSM 800 was used with the parameters as follow: 63x (NA_1.40); 1.3–1.6x optical zoom, averaged 2 times, scan speed 4–6, 0.35 μm or optimal interval with 16-bit dynamic range. Acquisition settings were kept consistent for all experimental groups within a given experiment, except for the GFP or RFP staining which was used to visualize dendritic arbors and spines.

For the analysis of fluorescence intensity and dendritic spine density, segments (60–90 μm for images taken by Olympus, LSM700 LSM780, and LSM 800; 35–60 μm for images taken by SIM) of two to three basal dendrites or secondary apical dendrites of a transfected neuron were analyzed from maximum-projected images. The density and dimension of individual spines, fluorescent intensity and co-localization were measured manually by using the MetaMorph software. Dendritic spines were categorized using previously described criteria [31]. Spines were defined as those having the ratio of head width to neck width > = 1.5. For an unbiased analysis, the quantification of spine density was performed blinded.

For quantitative analysis of synaptic puncta in CA1 striatum radiatum (SR) of hippocampus, the MetaMorph software was used to automatically outline the Homer1 puncta. The minimal width of puncta was set at 0.21–0.24 μm. The SR area of each image was outlined based on the DAPI staining. Density of Homer1 puncta in each image was calculated through dividing the number of puncta by the SR area. The experimenter who performed the synaptic quantification was blinded to the sample conditions.

For the quantification of colocalization overlap coefficient of TBC1D24 and Bassoon or PSD-95, the software ZEN2.3 (black) from Carl Zeiss was used. The puncta of TBC1D24 in dendritic spines were manually outlined as region of interest (ROI) to calculate the Mander’s overlap coefficient (MOC) [93].

### Behavior analysis

Behavioral experiments were performed with 12- to 13-weeks old mice. Open field test was conducted within a square open field (40×40×40 cm). Mice were placed individually in the center of the open field and allowed to freely explore for 15 min. The open field was divided into two zones: center (20×20 cm) and outer ring (10 cm). For each mouse, the total travel distance and the time spent in each zone were measured by a computerized tracking system (Any-maze, Stoelting). Before and after each trial, the field was cleaned thoroughly with ddH_2_O and 70% ethanol and dried with paper towels.

Fear conditioning test was performed subsequent to open field test with 48 hours interval. During conditioning, mice were placed in the conditioning chambers individually. They were allowed to freely explore the chamber for 60 seconds (pre-stimuli period). Thereafter, the auditory cue was presented as a conditioned stimulus (CS) (Frequency: 4000HZ) for 30 seconds, and a 0.5 mA foot shock was given to the mice as an unconditional stimulus (US) during the last 2 sec of the sound. The presentation of the CS-US paring was repeated three times per session (start at 60, 110, and 160 sec) with 20-second interval between CS-US stimuli to strengthen the association. Following the final foot shock, the mice were left undisturbed in the chambers for 60 sec (post-stimuli period). Mice were returned to its holding cage on the same day. Contextual fear recall test was conducted 24 hours after conditioning: mice were returned to the same chamber and recorded for 5 minutes without any stimulus. Freezing behavior was recorded by Freezeframe4 (Coulbourn), while the percentage of freezing time (%) was calculated for analysis. Before and after each trial, the chamber was carefully cleaned with ddH_2_O and 70% ethanol and dried with paper towels.

### PTZ induced seizure

Pentylenetetrazol (PTZ, Sigma-Aldrich) was dissolved in PBS at 5 mg/ml. After intraperitoneal injection with a single convulsive dose of 50 mg/kg, mouse was observed for seizures in a chamber that allowed exploration for 30 min, during which behavioral responses were recorded and seizures were classified according to the Racine scale [94]: 0) normal; 1) decreased motion and movement; 2) head-nodding, facial and forelimb clonus; 3) myoclonic jerks of the head and neck, with brief twitching movements, or repetitive movements with head bobbing or tail rigidity; 4) forelimb or forelimb and hind limb clonus, reciprocal forepaw padding, hind limb abduction, continuous rearing, and falling, Straub tail response; 5) tonic convulsions; 6) death. After the experiment, all mice were sacrificed.

### Statistical analysis

Data are presented as mean + SEM. The normal distribution of each set of data was determined by D’Agostino & Pearson omnibus test using GraphPad Prism software (version 6.01). When normality was achieved, statistical analysis was performed by unpaired Student’s *t*-test, one-way ANOVA followed by Tukey analysis, or two-way ANOVA followed by Sidak analysis. When the data was not normally distributed, the statistical analysis was performed using non-parametric Mann-Whitney test in experiments with two experimental conditions and Kruskal-Wallis test followed by Dunn’s multiple comparisons for experiments with three or more experimental conditions. Statistical significance was defined as p<0.05.

## Supporting information

S1 Fig Identification of excitatory and inhibitory neurons, and validation of knockdown efficiency by TBC1D24-shRNA. **(A)** Knockdown efficiency of the TBC1D24-shRNA was confirmed by Western blot analysis for lysate of cortical neurons transfected with TBC1D24-shRNA or control shRNA by nucleofection. The introduction TBC1D24-shRNA decreased the expression of TBC1D24 in neurons by more than 50%. **(B)** Immunostaining demonstrated that introduction of TBC1D24-shRNA reduced the expression of TBC1D24 and spine density in cultured hippocampal neurons. Neurons (12–13 DIV) were transfected by calcium phosphate precipitation and fixed at 18–19 DIV. Density of dendritic spines was quantified (25–33 dendrites from 12–17 neurons were quantified for each condition; results were pooled from two independent experiments; ****p<0.0001; unpaired Student’s *t* test). Representative images of TBC1D24 immunostaining on cell bodies (insets) were shown, and the intensity of TBC1D24 in cell body was quantified; mean+SEM; **p<0.01, unpaired Student’s *t*-test. Scale bars: top, 20 μm and bottom, 5 μm. **(C)** Hippocampal neurons were transfected with GFP at 15 DIV and fixed for immunofluorescence staining of GAD-67 three days post transfection. The percentage of neurons expressing GAD-67 in the cell body (yellow arrowhead) in culture was about 3% (24 out of 851 neurons examined from 24 fields) and none of the randomly picked GFP-positive neuron (red arrowhead) expressed GAD-67 in the soma. Scale bars: 30 μm.
(TIF)

S2 Fig Hipppocampal neurons expressing TBC1D24-shRNA in GFP-containing vector exhibit reduction of mushroom spine density *in vitro*. **(A)** Schematic diagram illustrating the constructs of control or TBC1D24-shRNA containing EGFP in the same plasmid **(B)** Hippocampal neurons were transfected with control-shRNA or TBC1D24-shRNA at 15 DIV and fixed at 3 days post transfection. The introduction of TBC1D24-shRNA significantly reduced the expression of TBC1D24 and the density of mushroom spine in GFP-positive neurons. Representative images of cell body (insets) and dendrites cropped from cells expressing control shRNA or TBC1D24-shRNA were shown. 12–18 dendrites from 8–11 neurons for each condition were quantified; *p<0.05; ****p<0.0001, unpaired Student *t* test. Scale bars: top, 20 μm; bottom, 5 μm.
(TIF)

S3 Fig TBC1D24 regulates dendritic spines and excitatory synapses via ARF6. **(A)** Hippocampal neurons (16 DIV) were co-transfected with GFP plasmid together with wild-type (WT) or various ARF6 mutants, followed by immunostaining three days post-transfection with vGLUT1 and PSD-95 antibodies. Neurons expressing constitutively-active ARF6 (ARF6-Q67L) exhibited a significant reduction of excitatory synapses on dendritic protrusions (31–37 dendrites from three independent experiments were quantified for each condition; mean+SEM; *p<0.05; Kruskal-Wallis test followed by Dunn’s multiple comparisons). Scale bar; 5 μm. **(B)** Hippocampal neurons (15 DIV) were co-transfected with GFP and TBC1D24-shRNA or control shRNA, followed by treatment with secinH3 (30 μM) or DMSO (as vehicle control) for 6 hours at 3 days post transfection. Treatment with secinH3 reversed the loss of excitatory synapses induced by TBC1D24-shRNA (24–30 dendrites from two independent experiments were quantified for each condition; *p<0.05, ****p<0.0001; Kruskal-Wallis test followed by Dunn’s multiple comparisons). Scale bar; 5 μm. **(C)** Hippocampal neurons (16 DIV) were co-transfected with GFP and control-shRNA in the presence or absence of wild-type (WT) or dominant-negative (T27N) ARF6. Neurons were fixed and immunostained with GFP antibody 3 days post transfection. The expression of wild-type or dominant-negative ARF6 did not significantly change the spine density (14–20 dendrites from two independent experiments). Scale bar: 10 μm.
(TIF)

S4 Fig Schematic diagram of TBC1D24 protein domains and DNA sequencing for disease-related TBC1D24 mutants. The Sanger sequencing confirmed correct nucleotide substitutions for the various TBC1D24 mutants.
(TIF)

S5 Fig The analyses of gross anatomy and migration of cortical neurons. **(A)** Representative images of bodies and whole brains from P20 wild-type and mutant mice were showed. The body size and whole-brain volume were comparable among three genotypes. Scale bars: left, 2 cm; right, 5 mm. **(B)** Brain sections from P20 wild-type and mutant mice were stained by antibody against NeuN. No defects in global structure and hippocampal morphology were observed in the mutant brains. Scale bars: left, 2 mm; right, 1 mm. **(C)** Brain sections from P20 mice were immunostained with DAPI, deep-layer cortical marker Tbr1, and upper-layer cortical marker Brn2. Heterozygous or homozygous F251L mutant mice demonstrated no abnormality in cortical development at P20. Scale bar: 100 μm.
(TIF)

S6 Fig The alignment of TBC1D24 protein in various species shows the affected amino acid Phe at position 251 is highly conserved. (TIF)

S1 Video Homozygous TBC1D24^F251L/F251L^ mice demonstrate lethal seizure attacks. The F251L homozygous (Hom) mouse (at P28) but not the wild-type littermate showed a sudden wild running and seizure followed by death. Wild-type, heterozygous and homozygous F251L knock-in mice at postnatal days 19–28 were monitored for seizure activities (three mice for each genotype). All three homozygous mice showed similar wild-running and convulsion right before they died, while none of the wild-type or heterozygous littermates display these behaviors and they did not die at these ages.
(MP4)

S1 Data Excel file containing numerical data used for all the figures in this study. (XLSX)
