# Supplementary figures and images for: The epilepsy and intellectual disability-associated protein TBC1D24 regulates the maintenance of excitatory synapses and animal behaviors
Source: PLoS Genet. 2020 Jan 31;16(1):e1008587. doi: 10.1371/journal.pgen.1008587 (PMC7015432; doi:10.1371/journal.pgen.1008587)

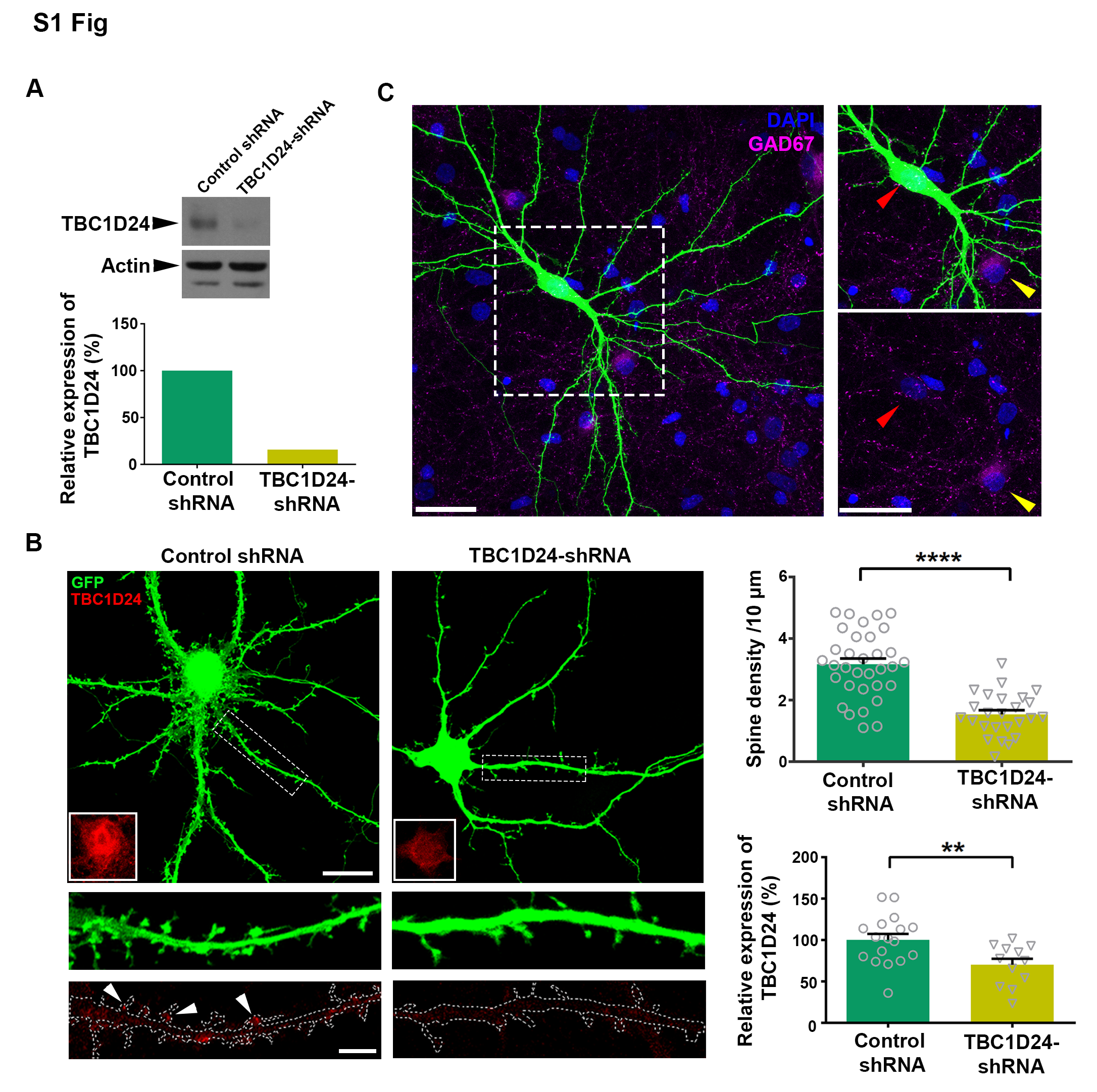

Supplement: S1 Fig — (A) Knockdown efficiency of the TBC1D24-shRNA was confirmed by Western blot analysis for lysate of cortical neurons transfected with TBC1D24-shRNA or control shRNA by nucleofection. The introduction TBC1D24-shRNA decreased the expression of TBC1D24 in neurons by more than 50%. (B) Immunostaining demonstrated that introduction of TBC1D24-shRNA reduced the expression of TBC1D24 and spine density in cultured hippocampal neurons. Neurons (12–13 DIV) were transfected by calcium phosphate precipitation and fixed at 18–19 DIV. Density of dendritic spines was quantified (25–33 dendrites from 12–17 neurons were quantified for each condition; results were pooled from two independent experiments; ****p<0.0001; unpaired Student’s t test). Representative images of TBC1D24 immunostaining on cell bodies (insets) were shown, and the intensity of TBC1D24 in cell body was quantified; mean+SEM; **p<0.01, unpaired Student’s t-test. Scale bars: top, 20 μm and bottom, 5 μm. (C) Hippocampal neurons were transfected with GFP at 15 DIV and fixed for immunofluorescence staining of GAD-67 three days post transfection. The percentage of neurons expressing GAD-67 in the cell body (yellow arrowhead) in culture was about 3% (24 out of 851 neurons examined from 24 fields) and none of the randomly picked GFP-positive neuron (red arrowhead) expressed GAD-67 in the soma. Scale bars: 30 μm. (TIF) [file pgen.1008587.s001.tif]

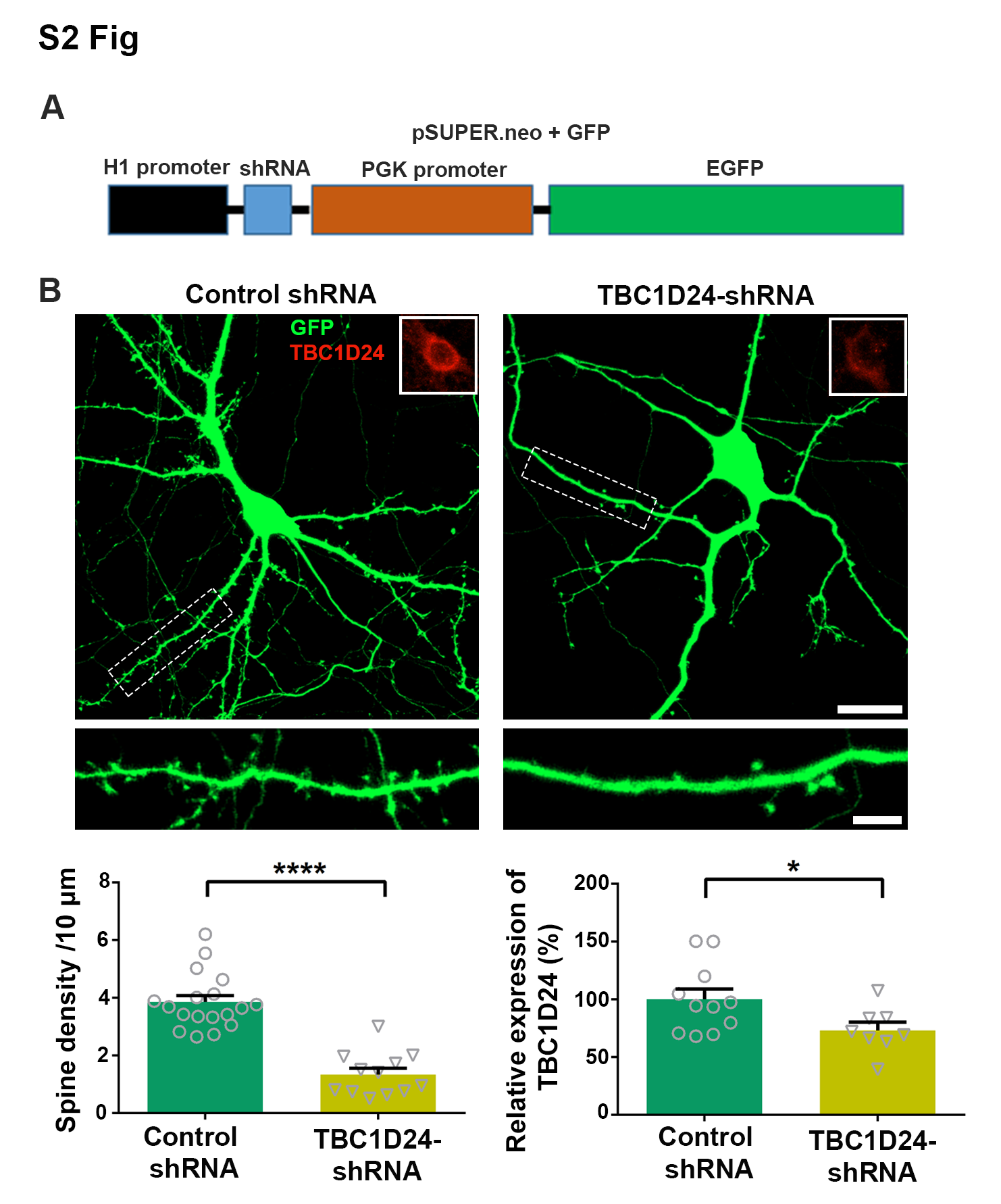

Supplement: S2 Fig — (A) Schematic diagram illustrating the constructs of control or TBC1D24-shRNA containing EGFP in the same plasmid (B) Hippocampal neurons were transfected with control-shRNA or TBC1D24-shRNA at 15 DIV and fixed at 3 days post transfection. The introduction of TBC1D24-shRNA significantly reduced the expression of TBC1D24 and the density of mushroom spine in GFP-positive neurons. Representative images of cell body (insets) and dendrites cropped from cells expressing control shRNA or TBC1D24-shRNA were shown. 12–18 dendrites from 8–11 neurons for each condition were quantified; *p<0.05; ****p<0.0001, unpaired Student t test. Scale bars: top, 20 μm; bottom, 5 μm. (TIF) [file pgen.1008587.s002.tif]

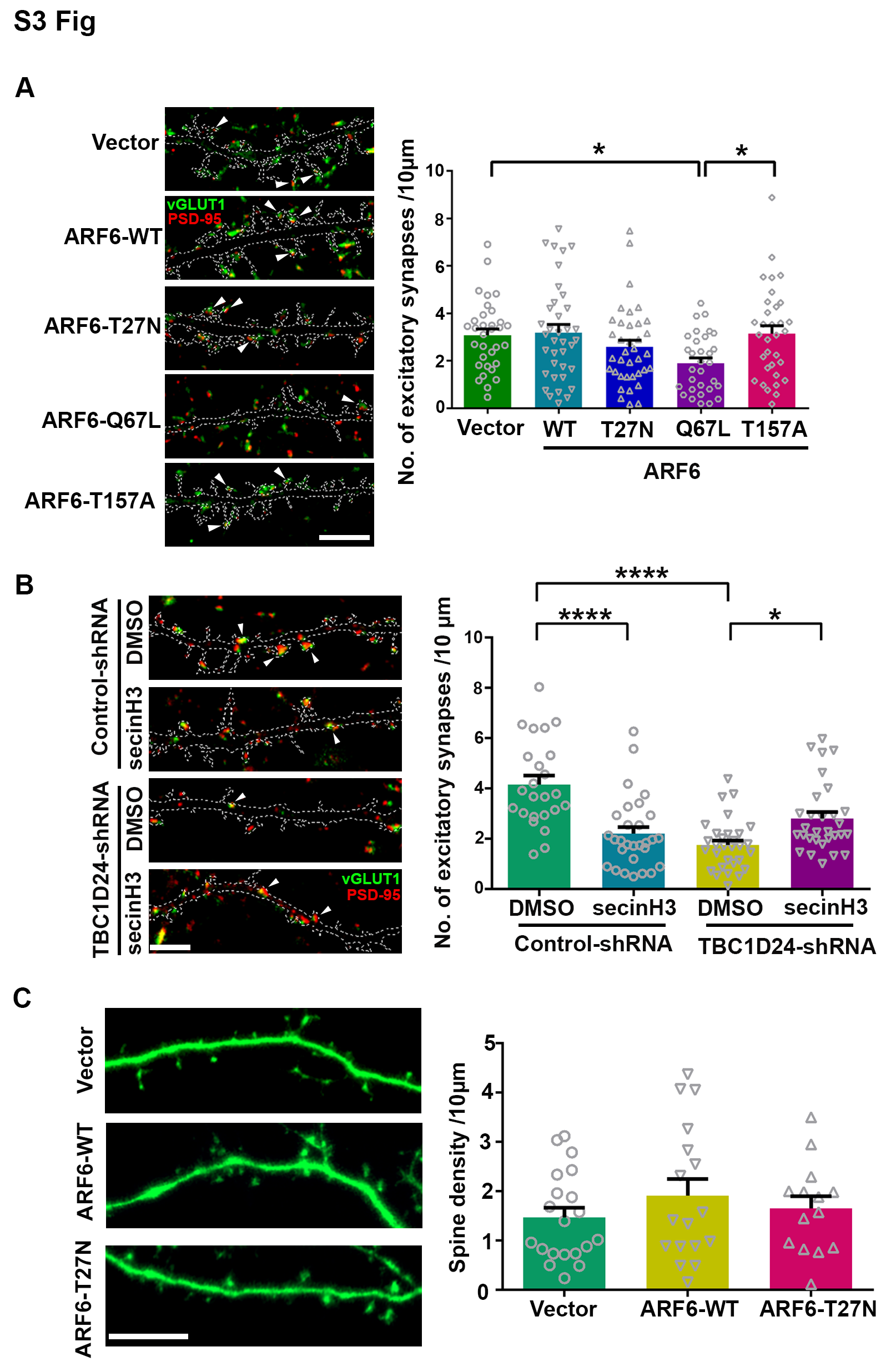

Supplement: S3 Fig — (A) Hippocampal neurons (16 DIV) were co-transfected with GFP plasmid together with wild-type (WT) or various ARF6 mutants, followed by immunostaining three days post-transfection with vGLUT1 and PSD-95 antibodies. Neurons expressing constitutively-active ARF6 (ARF6-Q67L) exhibited a significant reduction of excitatory synapses on dendritic protrusions (31–37 dendrites from three independent experiments were quantified for each condition; mean+SEM; *p<0.05; Kruskal-Wallis test followed by Dunn’s multiple comparisons). Scale bar; 5 μm. (B) Hippocampal neurons (15 DIV) were co-transfected with GFP and TBC1D24-shRNA or control shRNA, followed by treatment with secinH3 (30 μM) or DMSO (as vehicle control) for 6 hours at 3 days post transfection. Treatment with secinH3 reversed the loss of excitatory synapses induced by TBC1D24-shRNA (24–30 dendrites from two independent experiments were quantified for each condition; *p<0.05, ****p<0.0001; Kruskal-Wallis test followed by Dunn’s multiple comparisons). Scale bar; 5 μm. (C) Hippocampal neurons (16 DIV) were co-transfected with GFP and control-shRNA in the presence or absence of wild-type (WT) or dominant-negative (T27N) ARF6. Neurons were fixed and immunostained with GFP antibody 3 days post transfection. The expression of wild-type or dominant-negative ARF6 did not significantly change the spine density (14–20 dendrites from two independent experiments). Scale bar: 10 μm. (TIF) [file pgen.1008587.s003.tif]

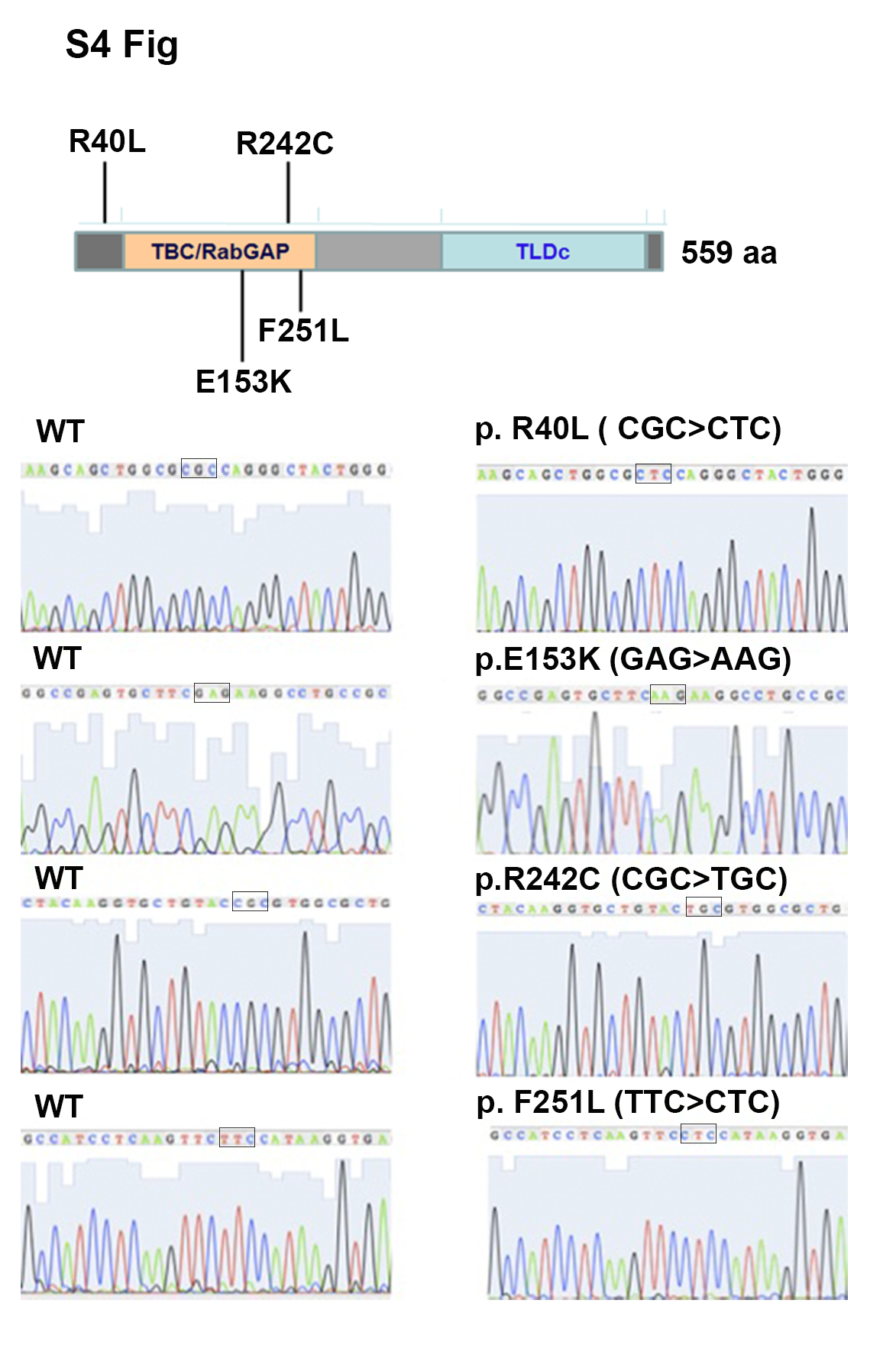

Supplement: S4 Fig — The Sanger sequencing confirmed correct nucleotide substitutions for the various TBC1D24 mutants. (TIF) [file pgen.1008587.s004.tif]

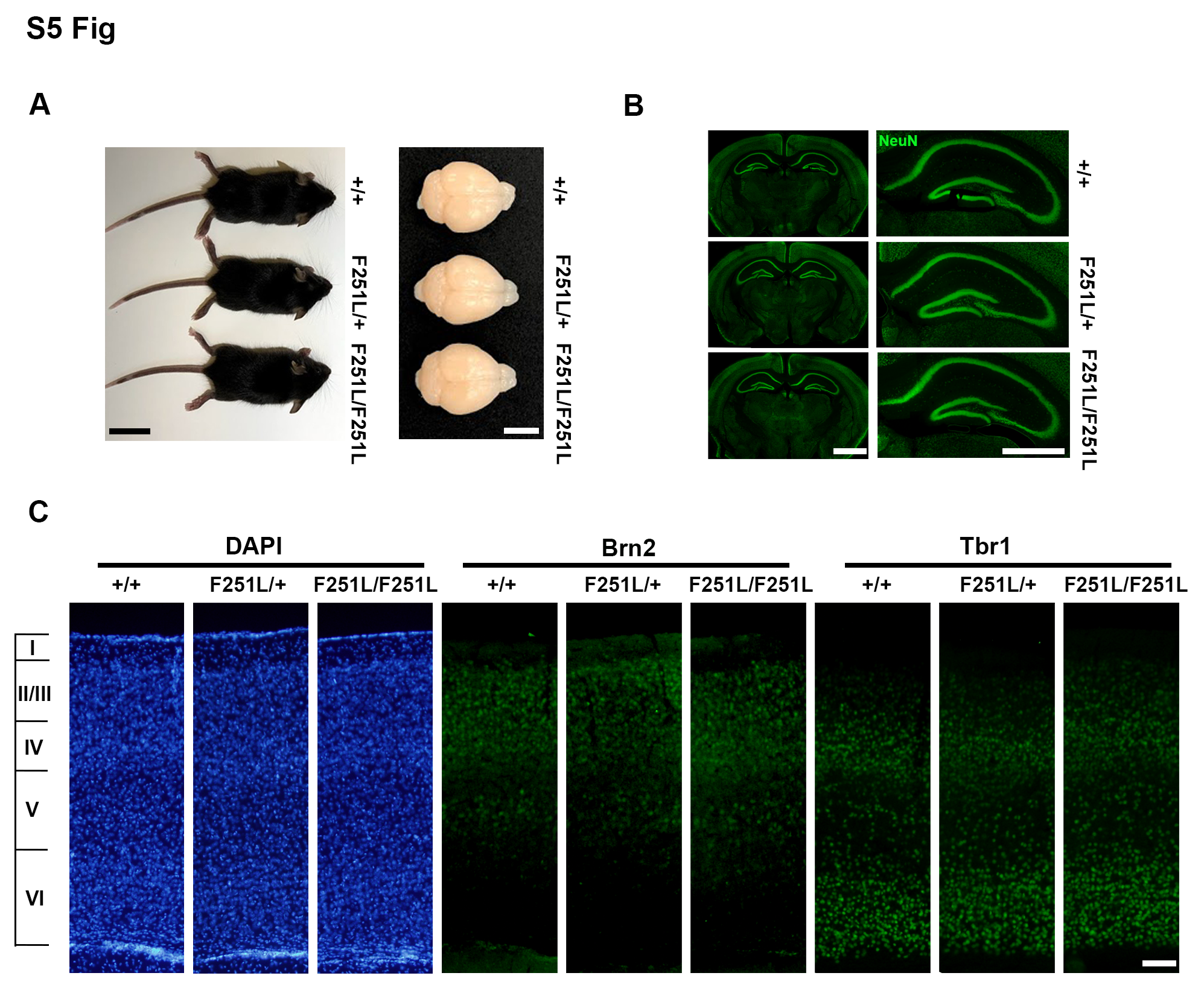

Supplement: S5 Fig — (A) Representative images of bodies and whole brains from P20 wild-type and mutant mice were showed. The body size and whole-brain volume were comparable among three genotypes. Scale bars: left, 2 cm; right, 5 mm. (B) Brain sections from P20 wild-type and mutant mice were stained by antibody against NeuN. No defects in global structure and hippocampal morphology were observed in the mutant brains. Scale bars: left, 2 mm; right, 1 mm. (C) Brain sections from P20 mice were immunostained with DAPI, deep-layer cortical marker Tbr1, and upper-layer cortical marker Brn2. Heterozygous or homozygous F251L mutant mice demonstrated no abnormality in cortical development at P20. Scale bar: 100 μm. (TIF) [file pgen.1008587.s005.tif]

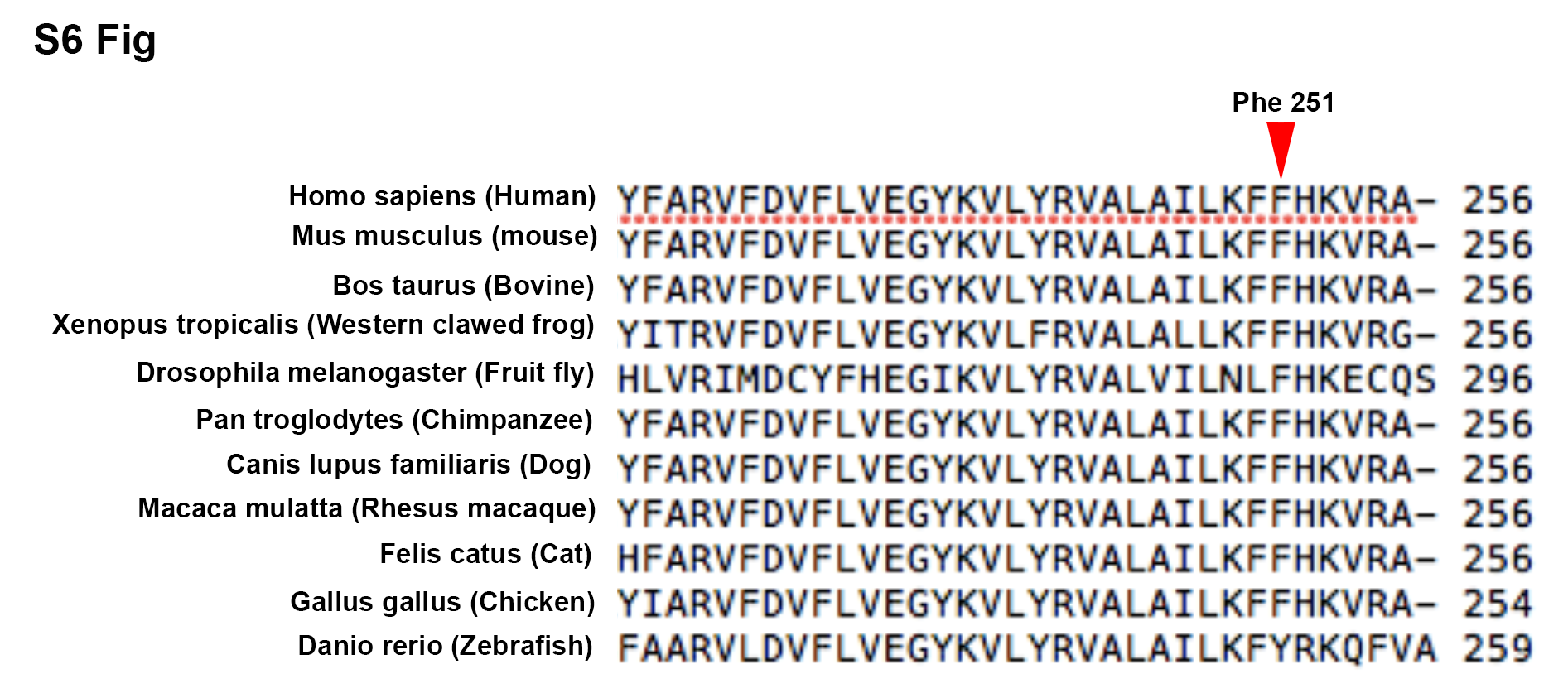

Supplement: S6 Fig — (TIF) [file pgen.1008587.s006.tif]
